# Mendelian randomization identifies proteins involved in neurodegenerative diseases

**DOI:** 10.1093/brain/awaf018

**Published:** 2025-03-03

**Authors:** Lazaros Belbasis, Sam Morris, Cornelia van Duijn, Derrick Bennett, Robin Walters

**Affiliations:** Nuffield Department of Population Health, University of Oxford, Oxford OX3 7LF, UK; Nuffield Department of Population Health, University of Oxford, Oxford OX3 7LF, UK; Nuffield Department of Population Health, University of Oxford, Oxford OX3 7LF, UK; Nuffield Department of Population Health, University of Oxford, Oxford OX3 7LF, UK; Nuffield Department of Population Health, University of Oxford, Oxford OX3 7LF, UK

**Keywords:** Alzheimer’s disease, amyotrophic lateral sclerosis, Mendelian randomization, multiple sclerosis, Parkinson’s disease, proteomics

## Abstract

Proteins are involved in multiple biological functions. High-throughput technologies have allowed the measurement of thousands of proteins in population biobanks. In this study, we aimed to identify proteins related to Alzheimer’s disease, Parkinson’s disease, multiple sclerosis and amyotrophic lateral sclerosis by leveraging large-scale genetic and proteomic data.

We performed a two-sample *cis* Mendelian randomization study by selecting instrumental variables for the abundance of >2700 proteins measured by either Olink or SomaScan platforms in plasma from the UK Biobank and the deCODE Health Study. We also used the latest publicly available genome-wide association studies for the neurodegenerative diseases of interest. The potentially causal effect of proteins on neurodegenerative diseases was estimated based on the Wald ratio.

We tested 13 377 protein–disease associations, identifying 169 associations that were statistically significant (5% false discovery rate). Evidence of co-localization between plasma protein abundance and disease risk (posterior probability > 0.80) was identified for 61 protein–disease pairs, leading to 50 unique protein–disease associations. Notably, 23 of 50 protein–disease associations corresponded to genetic loci not previously reported by genome-wide association studies. The two-sample Mendelian randomization and co-localization analysis also showed that APOE abundance in plasma was associated with three subcortical volumes (hippocampus, amygdala and nucleus accumbens) and white matter hyper-intensities, whereas PILRA and PILRB abundance in plasma was associated with caudate nucleus volume.

Our study provided a comprehensive assessment of the effect of the human proteome that is currently measurable through two different platforms on neurodegenerative diseases. The newly associated proteins indicated the involvement of complement (C1S and C1R), microglia (SIRPA, SIGLEC9 and PRSS8) and lysosomes (CLN5) in Alzheimer’s disease; the interleukin-6 pathway (CTF1) in Parkinson’s disease; lysosomes (TPP1), blood–brain barrier integrity (MFAP2) and astrocytes (TNFSF13) in amyotrophic lateral sclerosis; and blood–brain barrier integrity (VEGFB), oligodendrocytes (PARP1), node of Ranvier and dorsal root ganglion (NCS1, FLRT3 and CDH15) and the innate immune system (CR1, AHSG and WARS) in multiple sclerosis. Our study demonstrates how harnessing large-scale genomic and proteomic data can yield new insights into the role of the plasma proteome in the pathogenesis of neurodegenerative diseases.

## Teaser

Belbasis *et al.* identify 23 novel proteins linked to neurodegenerative diseases using genetic and proteomic data in a Mendelian randomisation framework. The findings highlight the role of the immune system, lysosomes, and blood–brain barrier integrity in the development of these diseases.

## Introduction

Neurological diseases are the leading cause of disability and the second leading cause of death worldwide.^1^ Neurodegenerative diseases constitute a distinct group of neurological diseases, which are characterized by progressive neuronal loss and formation of distinct pathological changes in the brain.^2^ During the last three decades, there has been a substantial increase in the number of people living with neurodegenerative diseases such as Alzheimer’s disease (AD), Parkinson’s disease (PD), amyotrophic lateral sclerosis (ALS) and multiple sclerosis (MS).^3-6^ Although MS is mainly an autoimmune disorder, early signs of neurodegeneration are evident in the disease course as a reactive response to the autoimmune process.^7^ Genome-wide association studies (GWAS) have identified molecular pathways leading to neurodegenerative diseases and have increased our knowledge on causal pathways involved in these diseases.^8-11^

Proteins play a key role in a range of biological processes, hence their dysregulation can lead to the development of diseases, and even minor modulation of their levels or function can modify disease risk. They represent a major source of biomarkers for the diagnosis or prediction of disease and can also be crucial to improving our understanding of the pathogenesis of diseases.^12^ About 75% of US Food and Drug Administration-approved medications were targeted at human proteins.^13,14^ Therefore, by combining large-scale genomic and proteomic profiling, there is a potential to identify disease-causing pathways, uncover new drug targets, highlight new therapeutic indications and identify clinically relevant biomarkers.^15,16^

Recent technological advances have allowed the measurement of thousands of proteins in large population-based studies. To date, two different high-throughput techniques to measure the abundance of multiple proteins have been used in large population samples: an antibody-based proximity-extension assay (Olink platform) and an aptamer affinity-based assay (SomaScan platform).^16^ GWAS of plasma protein abundance have identified protein quantitative trait loci (pQTLs), which can be used to examine the potentially causal effect of proteins on human diseases and traits using the Mendelian randomization (MR) framework.^12^ MR is an instrumental variable (IV) approach, which can be used to accelerate the discovery of biomarkers and the drug development pipeline.^17^ MR studies have examined the potential role of proteins in the development of neurological diseases, mainly by adopting a transcriptome-wide MR approach, which uses expression quantitative trait loci (eQTLs) as IVs^18-23^; however, the value of such analyses is limited by the fact that eQTLs frequently do not reflect protein abundance accurately.^24^

**Figure 1:**
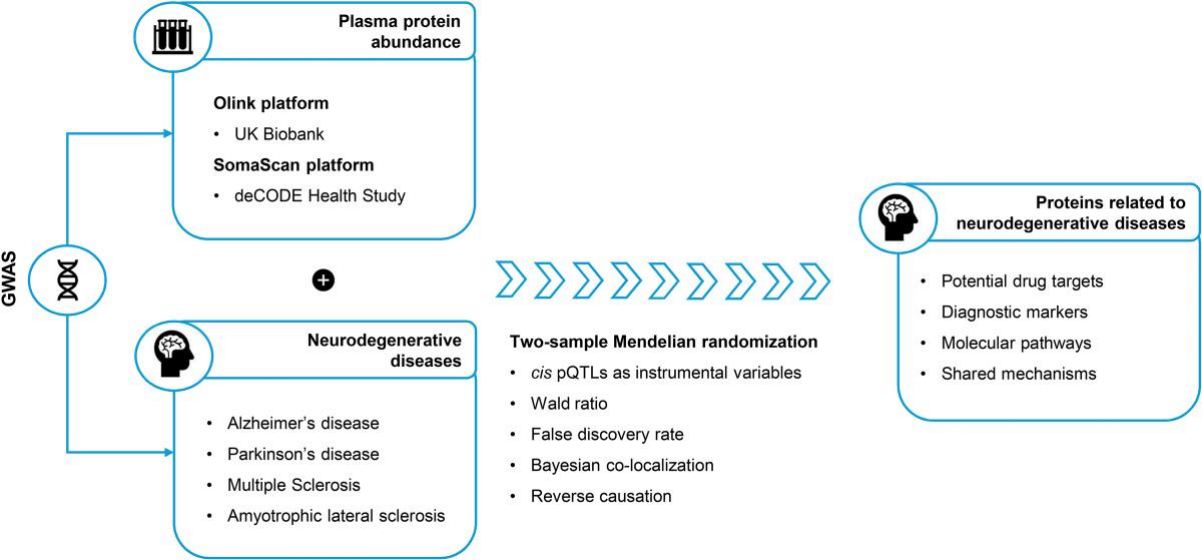
**Schematic representation of the study.**  *Cis* protein quantitative trait loci (pQTLs) from two large proteogenomic studies were used as instrumental variables for protein abundance in plasma. These were then integrated with genome-wide association studies (GWAS) for four neurodegenerative diseases. The association of plasma proteins with neurodegenerative diseases was assessed by estimating the Wald ratio within a two-sample Mendelian randomization framework. The analysis was complemented by Bayesian co-localization. The potentially causal proteins for neurodegenerative diseases might serve as drug targets and diagnostic markers or help to elucidate molecular pathways and shared biological mechanisms. The figure was created using Microsoft PowerPoint.

In the present study, we harnessed summary-level genetic data from two large proteo-genomic studies and from the largest GWAS for major neurodegenerative diseases, to identify proteins whose abundance in plasma is associated with these diseases (Fig. 1). We followed a two-sample *cis* MR approach, and we minimized the risk of confounding by linkage disequilibrium (LD) by performing a co-localization analysis.^25^ We complemented our analysis by exploring the potential effects of these proteins on multiple brain imaging phenotypes.

## Materials and methods

### Data sources

#### Genome-side association study of human plasma proteome

We used summary-level data from the two largest proteogenomics studies conducted in populations of European ancestry using either Olink or SomaScan platforms, which were identified through a publicly available catalogue of proteogenomics studies (last updated on 29 January 2024).^16^ The association between circulating protein levels and genetic variants was assessed in 35 571 participants in the UK Biobank using the Olink Explore 3072 platform, measuring 2941 protein analytes, capturing 2923 unique proteins,^26^ and in 35 559 participants in the Icelandic Cancer Project and deCODE Health Study using the SomaScan version 4 platform, measuring 4907 aptamers, capturing 4719 proteins.^27^

We do not assume equivalence of the two platforms, but we included pQTLs identified through both platforms to increase the completeness of our analysis.

#### Genome-wide association study of neurological diseases

We searched GWAS Catalog for published GWAS on neurological diseases (Supplementary Table 1).^28^ We selected the largest publicly available GWAS in population of European ancestry for AD (111 326 cases, 75 genome-wide significant variants),^9^ PD (33 674 cases, 90 genome-wide significant variants),^8^ MS (47 429 cases, 200 genome-wide significant variants)^11^ and ALS (27 205 cases, 15 genome-wide significant variants).^10^

### Selection of instrumental variables for protein abundance

pQTLs are genetic variants with an effect on protein expression, and they can be either *cis* or *trans* based on their proximity to the gene encoding the protein of interest.^29^  *trans* pQTLs map to genes that do not code directly for the targeted proteins or that correspond to intergenic regions, and it is difficult to distinguish between detected effects owing to vertical and horizontal pleiotropy.^12,30^ For this reason, we restricted our MR analysis to *cis* pQTLs. Only autosomal genetic variants were included in the analyses, because summary-level data for the X chromosome are not available in some of the GWAS for neurological diseases.

We retrieved statistically significant *cis* pQTLs from each of the proteogenomics studies, applying the same level of statistical significance as used in those studies (*P* < 3.40 × 10^−11^ for the study using the Olink platform, and *P* < 1.80 × 10^−9^ for the study using the SomaScan platform). For the Olink platform, the *cis* region was defined as a distance of 1 Mb upstream or downstream from the end or start, respectively, of the gene encoding the protein of interest.^26^ For the SomaScan platform, the *cis* region was defined as a distance of 1 Mb upstream or downstream from the transcription start site of the gene encoding the protein of interest.^27^

We used the following criteria to filter the list of statistically significant *cis* pQTLs in each study:

- Owing to the complex LD structure of single nucleotide polymorphisms within the human major histocompatibility complex region, single nucleotide polymorphisms and proteins encoded by genes within the major histocompatibility complex region (Chromosome 6: from 26 to 34 Mb) were excluded.
- To reduce the risk for weak instrument bias, we calculated the *F*-statistic for each single nucleotide polymorphism, and we excluded genetic instruments with an *F*-statistic of <10.^31^
- We obtained the genetic variants that were also tested in the GWAS for neurological diseases.
- For each protein, we selected only the *cis* pQTL with the lowest *P*-value, which we refer as the ‘lead variant’ or ‘lead *cis* pQTL’.

There is substantial heterogeneity between Olink and SomaScan platforms, which means that they might measure different proteoforms of the same protein.^32^ For this reason, when an assay in both platforms targeted the same protein, we considered the lead *cis* pQTL from both platforms. When multiple assays in the same platform targeted the same protein (as defined by UniProt ID), we included only the instrument with the lowest *P*-value.

### Data harmonization

All GWAS summary statistics were lifted over to genomic build 38.^33^ We followed the recommended harmonization framework for two-sample MR analyses.^34,35^ Ambiguous palindromic single nucleotide polymorphisms with an allele frequency between 0.42 and 0.58 were excluded to avoid potential allele mismatch across different GWAS.^34^ Data harmonization was implemented using the *TwoSampleMR* package.^36,37^

### Statistical analysis

#### Association of protein abundance with neurological diseases

The Wald ratio, which is defined as the ratio of the gene-outcome effect divided by the gene-exposure effect, was calculated for all the protein–disease associations.^38^ To identify the statistically significant associations, a multiplicity correction was applied using the Benjamini–Hochberg method.^39^ Evidence of a statistically significant protein–disease association was based on 5% false discovery rate (FDR). MR analyses were performed using the *TwoSampleMR* package.^36,37^ We prioritized proteins with a statistically significant association with a neurological disease for further analyses to: (i) assess reverse causality; and (ii) perform Bayesian co-localization.

#### Assessment of reverse causation

Reverse causation could be a potential explanation for positive findings in an MR analysis. We explored the potential for reverse causality by taking a bi-directional MR approach. We performed LD clumping to obtain approximately independent genetic variants to model the genetic liability to AD, PD, ALS and MS. Clumping was performed using the reference panel from 1000Genomes for population of European ancestry, setting a statistical significance threshold of *P* < 5 × 10^−8^, a genetic window of 1 Mb and an LD *r*^2^ < 0.1%. We used the *ld_clump* function from the *ieugwas* package and PLINK version 1.90.^40^ We derived four genetic instruments consisting of genome-wide significant genetic variants as reported in the relevant publications.^8-11^ We used these genetic instruments to examine whether the genetically predicted liability to each one of the neurological diseases of interest was related to the proteins associated with each one of the diseases. We estimated the Wald ratio for each one of the genetic variants, and we combined them using a random-effects inverse-variance weighted model.^41^ Evidence of statistically significant findings were based on 5% FDR.

#### Bayesian co-localization

Evidence of co-localization supports the validity of the IVs and strengthens the MR findings.^25^ To assess potential confounding by LD, we examined whether the prioritized proteins share the genetic variant with the outcomes of interest by conducting a co-localization analysis assuming a single causal variant in each genetic locus.^42^ We used the *coloc* package for the co-localization analysis. Variants within ±1 Mb window around the *cis* pQTLs with the smallest *P*-value were included. We used a posterior probability of >80% as strong evidence of co-localization, and a posterior probability of >60% as moderate evidence of co-localization. However, we acknowledge that lack of co-localization does not invalidate the MR findings, because co-localization methods have a high false negative rate.^13,43^ The GWAS by Bellenguez *et al*.^9^ for AD does not provide the majority of the genetic variants in the *APOE* gene locus. For this reason, we repeated the co-localization analysis using the GWAS by Kunkle *et al*.^44^ for the genetic loci located near *APOE* (i.e. *APOE*, *APOC1* and *NECTIN2*).

#### Association of mRNA abundance with neurological diseases

GWAS of gene expression reported *cis* eQTLs that are genetic variants affecting the mRNA abundance.^45^ The eQTLGen consortium examined eQTLs from blood-derived expression of 19 250 autosomal genes and reported at least one *cis* eQTL for 16 987 genes using a sample of 31 684 individuals.^46^ The MetaBrain consortium provides *cis* eQTLs in five tissues [cortex (2683 individuals), cerebellum (492 individuals), basal ganglia (208 individuals), hippocampus (168 individuals) and spinal cord (108 individuals)].^47^ For each one of the proteins measured through either Olink or SomaScan platforms, the relevant lead *cis* eQTL was selected as a genetic IV. We used the same statistical significance threshold as the GWAS on plasma and brain eQTLs to identify appropriate genetic instruments. We estimated the Wald ratio as the ratio of the genetic effect on disease risk divided by the genetic effect on mRNA abundance. A multiplicity correction was applied using the Benjamini–Hochberg method separately in plasma and brain tissues,^39^ and statistically significant associations were assessed at 5% FDR.

#### Association of protein abundance with brain imaging phenotypes

The potentially causal effect of the prioritized proteins on brain imaging traits was examined using summary-level GWAS data for nine brain volumes, mean cortical thickness and surface, and white matter hyper-intensities.^48-50^ The available brain volumes were intracranial volume,^51^ hippocampal volume^52^ and other subcortical structures volume^53^ (nucleus accumbens, amygdala, brainstem, caudate nucleus, globus pallidus, putamen and thalamus). A multiplicity correction was applied using the Benjamini–Hochberg method,^39^ and statistically significant findings were assessed at 5% FDR. Additionally, for the statistically significant associations, we performed Bayesian co-localization analysis assuming a single causal variant and using the same specifications as described before.

#### Downstream analyses

For the proteins that showed a statistically significant association with a neurodegenerative disease and had additional support from co-localization, we performed an enrichment analysis in Gene Ontology,^54^ Reactome Pathway Database,^55^ WikiPathways^56^ and Kyoto Encyclopedia of Genes and Genomes.^57^ A multiplicity correction was performed, and statistically significant enrichment was based on 5% FDR. We used the STRING database to perform the enrichment analysis.^58^ We also examined whether the identified proteins show high specificity in particular tissues or single cells, and whether they belong to a particular gene expression cluster using the Human Protein Atlas.^59,60^

## Results

### Association of plasma protein abundance with neurodegenerative diseases

**Figure 2:**
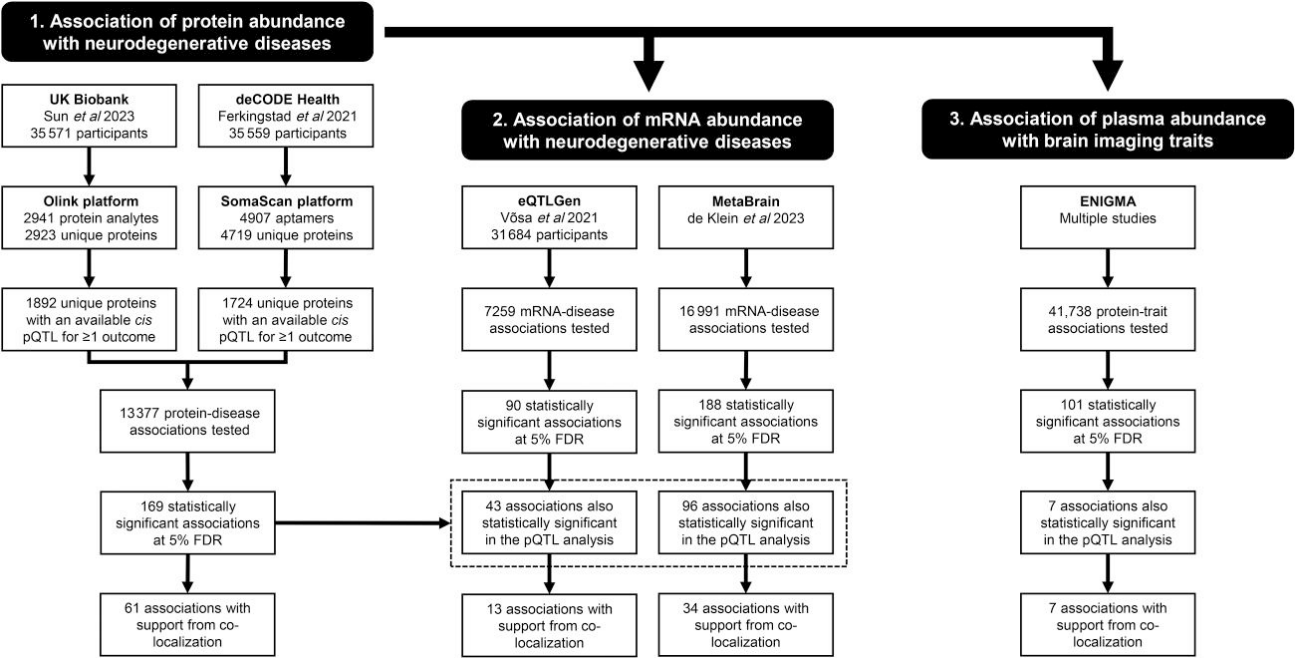
**Overview of the study design and main results.** The study consists of three key steps. First, the lead *cis* protein quantitative trait loci (pQTLs) from two proteogenomic genome-wide association studies (GWAS) using either the Olink or SomaScan platforms in European populations were used as instrumental variables (IVs) to perform a two-sample Mendelian randomization (MR) analysis for Alzheimer’s disease (AD), Parkinson’s disease (PD), multiple sclerosis (MS) and amyotrophic lateral sclerosis (ALS). A total of 13 377 protein–disease associations were tested, with 169 statistically significant associations at 5% false discovery rate (FDR) and 61 associations supported by co-localization. Second, MR analysis was repeated using plasma and brain *cis* eQTLs as IVs from the eQTLGen and MetaBrain consortia, yielding 43 (*eQTLGen*) and 96 (*MetaBrain*) overlapping associations with the pQTL analysis, with 13 and 34 supported by co-localization, respectively. Third, the association of plasma protein abundance with 12 brain-imaging traits was assessed using data from the ENIGMA consortium, identifying 101 statistically significant associations at 5% FDR, of which 7 were supported by co-localization.

**Figure 3:**
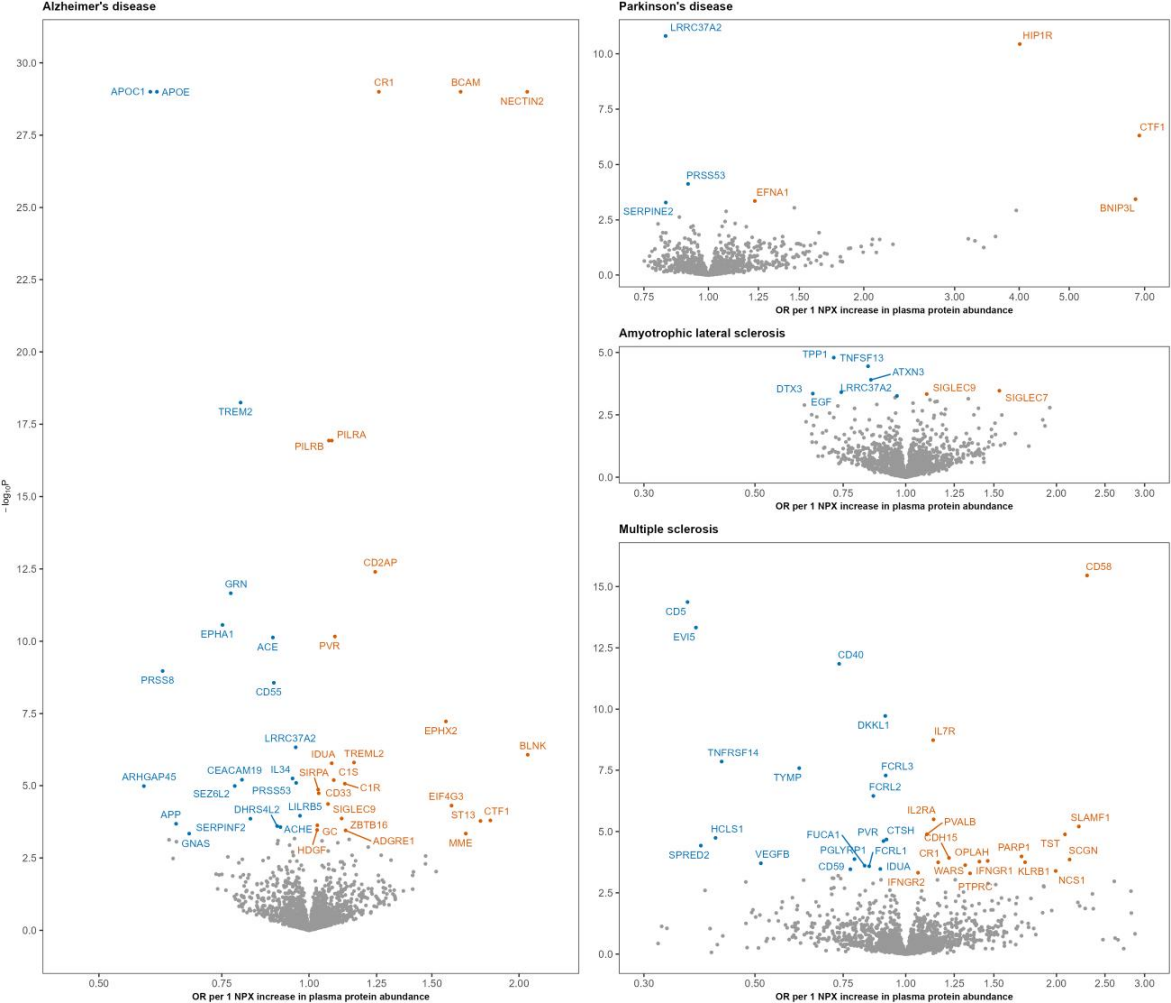
**Association of Olink proteins with neurodegenerative diseases using two-sample Mendelian randomization.** The lead *cis* protein quantitative trait locus was used as an instrumental variable (IV) for proteins measured through the Olink platform. In total, we tested 6762 protein–disease associations, and 95 of these were statistically significant (at 5% false discovery rate) and are annotated in this figure. The odds ratio corresponds to the Wald ratio, which is calculated by dividing the genetic effect of the IV on the disease by the genetic effect of the IV on the plasma protein abundance. To improve figure readability, one statistically significant association (TNFRSF1A with multiple sclerosis) with an extreme odds ratio (OR) is not shown. Additionally, in Alzheimer’s disease, five associations (APOE, APOC1, NECTIN2, BCAM and CR1) were capped at a −log_10_  *P* of 30 to enhance visual clarity.

**Figure 4:**
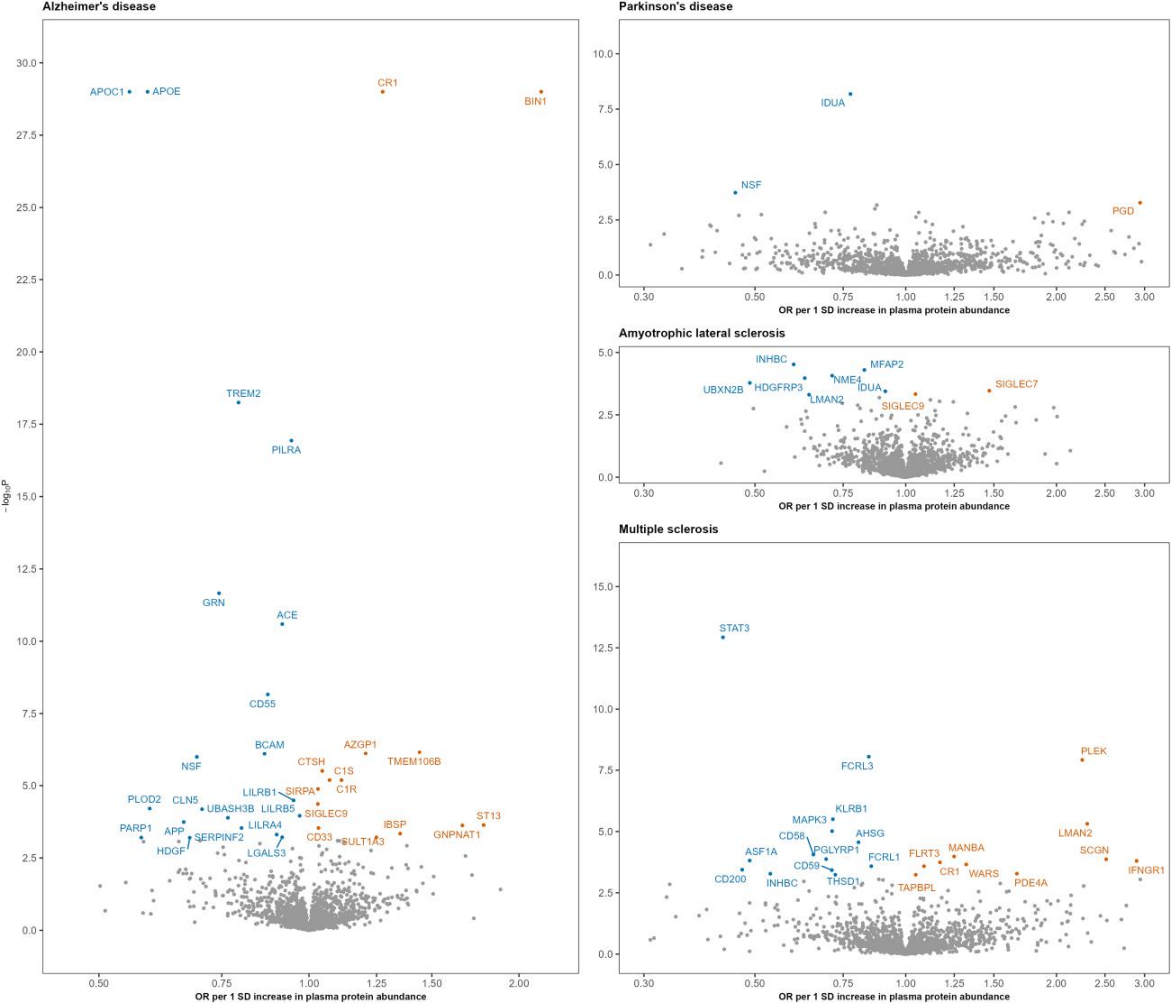
**Association of SomaScan proteins with neurodegenerative diseases using two-sample Mendelian randomization.** The lead *cis* protein quantitative trait locus was used as an instrumental variable (IV) for proteins measured through the SomaScan platform. In total, we tested 6615 protein–disease associations, and 74 of these were statistically significant (at 5% false discovery rate) and are annotated. The odds ratio (OR) corresponds to the Wald ratio, which is calculated by dividing the genetic effect of the IV on the disease by the genetic effect of the IV on the plasma protein abundance. To improve figure readability, five statistically significant associations (TNFRSF1A, DKKL1, JUND, IL2RA and PTPRJ with multiple sclerosis) with extreme ORs are not shown. Additionally, in Alzheimer’s disease, four associations from the SomaScan platform (APOE, APOC1, CR1 and BIN1) were capped at a −log_10_  *P* of 30 to enhance visual clarity.

An overview of the study design is presented in Fig. 2. To evaluate systematically the evidence for a causal effect of 2738 proteins on four neurological diseases (AD, PD, ALS and MS), we undertook a proteome-wide two-sample MR. All the selected IVs had an *F*-statistic > 10, minimizing the influence of weak instrument bias on the MR estimates. Overall, 13 377 protein–disease associations were tested (50.5% using *cis* pQTLs derived using Olink platform measurements and 49.5% using *cis* pQTLs from the SomaScan platform; Table 1 and Supplementary Table 2). We observed 1279 (9.6%) nominally significant protein–disease associations at *P* < 0.05, constituting a substantial excess in comparison to the number expected under the null. Of these, 169 protein–disease associations (1.3%) remained statistically significant at 5% FDR, corresponding to *P* < 6.3 × 10^−4^ (Tables 2 and 3 and Figs 3 and 4). Even after exclusion of these associations, the remaining associations displayed substantial inflation in comparison to the null (Table 1).

**Table 1:** Summary of the proteome-wide two-sample Mendelian randomization analysis on neurological diseases

Disease	Number of associations tested	Number of statistically significant associations				Inflation factor^b^	Co-localization^c^
		*P* < 0.05		5% FDR^a^			
Alzheimer’s disease	3610	420	(11.6%)	78	(2.2%)	1.36	27
Parkinson’s disease	2757	204	(7.4%)	10	(0.4%)	1.07	2
Amyotrophic lateral sclerosis	3518	278	(7.9%)	17	(0.5%)	1.28	3
Multiple sclerosis	3492	377	(10.8%)	64	(1.8%)	1.41	29
Total	13 377	1279	(9.6%)	169	(1.3%)	1.28	61

^a^False discovery rate based on Benjamini–Hochberg correction, which corresponds to *P* < 6.3 × 10^−4^.

^b^Inflation factor estimated after removal of the statistically significant associations at 5% false discovery rate (FDR).

^c^Posterior probability >80%.

**Table 2:** **Olink proteins associated with risk of neurodegenerative diseases identified using two-sample Mendelian randomization (5% FDR) and supported by co-localization (posterior probability > 0**.**80)**

Outcome	Assay ID^a^	Protein	rsID	EA/OA^b^	EAF	OR (95% CI)^c^	*P*-value	Novel^d^
AD	OID30727	APOE	rs8106813	G/A	0.494	0.61 (0.57–0.64)	3.37 × 10^−65^	No
	OID30697	CR1	rs679515	T/C	0.174	1.26 (1.21–1.31)	5.65 × 10^−33^	No
	OID20197	PILRB	rs1859788	G/A	0.681	1.07 (1.05–1.08)	1.16 × 10^−17^	No
	OID21129	PILRA	rs1859788	G/A	0.681	1.08 (1.06–1.10)	1.16 × 10^−17^	No
	OID20177	CD2AP	rs1385742	A/T	0.355	1.24 (1.17–1.32)	3.99 × 10^−13^	No
	OID21159	GRN	rs5848	C/T	0.726	0.77 (0.72–0.83)	2.19 × 10^−12^	No
	OID20763	PRSS8	rs889555	T/C	0.282	0.62 (0.53–0.72)	1.07 × 10^−9^	Yes
	OID30541	BLNK	rs55769428	A/C	0.962	2.06 (1.54–2.74)	8.55 × 10^−7^	No
	OID20809	IL34	rs4985556	C/A	0.878	0.95 (0.93–0.97)	5.65 × 10^−6^	No
	OID30731	C1S	rs12146727	G/A	0.865	1.09 (1.05–1.12)	6.41 × 10^−6^	Yes
	OID30753	C1R	rs10849546	G/A	0.865	1.13 (1.07–1.19)	8.55 × 10^−6^	Yes
	OID20304	SIRPA	rs6136377	A/G	0.618	1.03 (1.02–1.04)	1.37 × 10^−5^	Yes
	OID21472	CD33	rs2455069	G/A	0.427	1.03 (1.02–1.05)	1.83 × 10^−5^	No
	OID21390	SIGLEC9	rs2075803	A/G	0.447	1.06 (1.03–1.10)	4.30 × 10^−5^	Yes
	OID21205	ZBTB16	rs73000929	A/G	0.037	1.11 (1.05–1.18)	1.37 × 10^−4^	Yes
	OID21307	MME	rs79837905	A/G	0.920	1.68 (1.26–2.24)	4.54 × 10^−4^	No
PD	OID31141	HIP1R	rs10847864	T/G	0.359	4.01 (2.66–6.05)	3.64 × 10^−11^	No
	OID20061	CTF1	rs11150601	A/G	0.628	6.84 (3.23–14.46)	4.92 × 10^−7^	Yes
ALS	OID20750	TPP1	rs11827437	C/T	0.370	0.72 (0.62–0.83)	1.61 × 10^−5^	Yes
	OID20733	TNFSF13	rs3803800	A/G	0.211	0.84 (0.77–0.91)	3.59 × 10^−5^	Yes
MS	OID20716	CD58	rs10801908	C/T	0.880	2.30 (1.88–2.81)	3.54 × 10^−16^	No
	OID21449	CD5	rs4939491	G/A	0.609	0.37 (0.29–0.47)	4.28 × 10^−15^	No
	OID30519	EVI5	rs11808092	C/A	0.745	0.38 (0.30–0.49)	4.70 × 10^−14^	No
	OID21155	TNFRSF1A	rs1800693	T/C	0.597	0.12 (0.07–0.21)	1.02 × 10^−13^	No
	OID20724	CD40	rs4810485	G/T	0.752	0.74 (0.68–0.80)	1.41 × 10^−12^	No
	OID21313	DKKL1	rs2303759	T/G	0.748	0.91 (0.88–0.94)	1.89 × 10^−10^	No
	OID21136	IL7R	rs6897932	C/T	0.729	1.13 (1.09–1.18)	1.84 × 10^−9^	No
	OID20234	TYMP	rs131805	C/T	0.783	0.61 (0.52–0.73)	2.61 × 10^−8^	No
	OID20496	SLAMF1	rs7535367	G/T	0.861	2.22 (1.57–3.13)	6.24 × 10^−6^	No
	OID21420	PVALB	rs4821544	T/C	0.697	1.10 (1.05–1.15)	1.31 × 10^−5^	Yes
	OID20868	TST	rs4821544	T/C	0.697	2.08 (1.50–2.89)	1.31 × 10^−5^	Yes
	OID21011	PVR	rs2301274	T/C	0.759	0.90 (0.86–0.95)	2.46 × 10^−5^	No
	OID30423	SPRED2	rs7569084	C/T	0.414	0.39 (0.25–0.61)	3.76 × 10^−5^	No
	OID20500	PARP1	rs1433574	A/C	0.839	1.70 (1.30–2.23)	1.04 × 10^−4^	Yes
	OID21005	CDH15	rs11646135	A/G	0.142	1.22 (1.10–1.35)	1.19 × 10^−4^	Yes
	OID30697	CR1	rs679515	T/C	0.174	1.16 (1.07–1.26)	1.81 × 10^−4^	Yes
	OID30554	VEGFB	rs660442	A/G	0.200	0.51 (0.36–0.73)	1.98 × 10^−4^	Yes
	OID21084	WARS	rs12882934	C/A	0.745	1.31 (1.14–1.52)	2.36 × 10^−4^	Yes
	OID21458	NCS1	rs1054879	A/G	0.508	1.99 (1.36–2.92)	4.06 × 10^−4^	Yes

AD = Alzheimer’s disease; ALS = amyotrophic lateral sclerosis; CI = confidence interval; EA = effect allele; FDR = false discovery rate; MS = multiple sclerosis; OA = other allele; OR = odds ratio; PD = Parkinson’s disease.

^a^Assay identifier as provided by the Olink platform.

^b^The alleles have been orientated to reflect an increase in plasma protein abundance.

^c^The odds ratio correspond to risk for neurological disease per one normalized protein expression unit increase in plasma protein abundance measured through the Olink platform and estimated using the Wald ratio method.

^d^A genetic locus was considered novel if it was not reported by a genome-wide association study in European ancestry in the GWAS Catalog.

**Table 3:** **SomaScan proteins associated with risk of neurodegenerative diseases identified using two-sample Mendelian randomization (5% FDR) and supported by co-localization (posterior probability > 0**.**80)**

Outcome	Assay ID^a^	Protein	rsID	EA/OA^b^	EAF	OR (95% CI)^c^	*P*-value	Novel^d^
AD	19556_12	CR1	rs679515	T/C	0.183	1.28 (1.23–1.33)	5.65 × 10^−33^	No
	10816_150	PILRA	rs1859788	A/G	0.304	0.94 (0.93–0.96)	1.16 × 10^−17^	No
	4992_49	GRN	rs5848	C/T	0.744	0.74 (0.68–0.81)	2.19 × 10^−12^	No
	8687_26	TMEM106B	rs3173615	C/G	0.598	1.44 (1.25–1.66)	6.93 × 10^−7^	No
	8465_52	CTSH	rs2289702	C/T	0.894	1.04 (1.03–1.06)	3.10 × 10^−6^	No
	3285_23	C1R	rs12146727	G/A	0.881	1.11 (1.06–1.17)	6.41 × 10^−6^	Yes
	8840_61	C1S	rs12146727	G/A	0.881	1.07 (1.04–1.10)	6.41 × 10^−6^	Yes
	5430_66	SIRPA	rs6136376	A/G	0.640	1.03 (1.02–1.04)	1.30 × 10^−5^	Yes
	3007_7	SIGLEC9	rs2075803	A/G	0.479	1.03 (1.02–1.04)	4.30 × 10^−5^	Yes
	6923_1	PLOD2	rs148118826	G/A	0.997	0.59 (0.46–0.76)	6.18 × 10^−5^	Yes
	8874_53	CLN5	rs700363	G/A	0.910	0.70 (0.59–0.84)	6.51 × 10^−5^	Yes
ALS	9294_45	MFAP2	rs3738814	A/G	0.584	0.83 (0.75–0.91)	5.03 × 10^−5^	Yes
MS	2654_19	TNFRSF1A	rs1800693	T/C	0.597	0.19 (0.13–0.30)	1.02 × 10^−13^	No
	10346_5	STAT3	rs4796791	C/T	0.589	0.43 (0.35–0.54)	1.17 × 10^−13^	No
	16309_30	DKKL1	rs2288481	G/A	0.779	0.13 (0.07–0.26)	9.33 × 10^−10^	No
	4440_15	FCRL3	rs7528684	G/A	0.416	0.84 (0.80–0.89)	8.93 × 10^−9^	No
	9468_8	LMAN2	rs4131289	A/G	0.379	2.30 (1.61–3.30)	4.85 × 10^−6^	No
	3581_53	AHSG	rs4686790	T/G	0.707	0.80 (0.73–0.89)	2.74 × 10^−5^	Yes
	18172_71	ASF1A	rs4946366	T/C	0.164	0.49 (0.34–0.71)	1.53 × 10^−4^	Yes
	19556_12	CR1	rs679515	T/C	0.183	1.17 (1.08–1.27)	1.81 × 10^−4^	Yes
	9870_17	WARS	rs4905957	T/C	0.764	1.32 (1.14–1.53)	2.21 × 10^−4^	Yes
	13123_3	FLRT3	rs1932953	T/G	0.270	1.09 (1.04–1.14)	2.60 × 10^−4^	Yes

AD = Alzheimer’s disease; ALS = amyotrophic lateral sclerosis; CI = confidence interval; EA = effect allele; FDR = false discovery rate; MS = multiple sclerosis; OA = other allele; OR = odds ratio.

^a^Assay identifier as provided by the SomaScan platform.

^b^The alleles have been orientated to reflect an increase in plasma protein abundance.

^c^The odds ratio corresponds to the risk for neurological disease per one standard deviation increase in plasma protein abundance measured through SomaScan platform and estimated using the Wald ratio method.

^d^A genetic locus was considered novel if it was not reported by a genome-wide association study in European ancestry in the GWAS Catalog.

In our study, 120 unique proteins were associated with at least one neurodegenerative disease. Ten of them were associated with more than one neurodegenerative disease (Supplementary Figs 1 and 2). Also, 59 unique proteins were associated with AD, and 10 of them (17%) showed an association with AD and an additional neurological disease (CTF1, NSF and PRSS53 with PD; SIGLEC9 with ALS; CR1, CTSH, PARP1 and PVR with MS; LRRC37A2 with both PD and ALS; and IDUA with PD, ALS and MS). Two proteins (LMAN2 and INHBC) showed an association with both MS and ALS. However, we should highlight that the proteoform of IDUA measured through the Olink platform (OID21468) was associated with both AD and MS, whereas the proteoform of IDUA measured through the SomaScan platform (3169_70) was associated with both ALS and PD. The proteoform of CTSH measured through the Olink platform (OID20113) was associated with MS, and the proteoform of CTSH measured through the SomaScan platform (8465_52) was associated with AD.

Associations between proteins and phenotypes in the MR framework might reflect causality, but potential alternative explanations are reverse causality, confounding by LD or horizontal pleiotropy.^12^ We evaluate each of these explanations below.

### Cross-platform comparison of *cis* protein quantitative trait locus associations

We hypothesize that a consistent causal effect in both Olink and SomaScan platforms strengthens confidence in the instruments and in the robustness of the protein–disease associations, by showing that the inferred effect on disease risk is independent of the platform used for protein abundance measurement and pQTL identification. Indeed, 34 protein–disease associations were statistically significant across both platforms, but 5 of the 34 associations were not directionally consistent (BCAM, HDGF and PILRA for AD, and CD58 and KLRB1 for MS). Fourteen protein–disease associations were statistically significant only in the Olink platform (ARHGAP45, EPHA1, GC, IDUA, TREML2 and PRSS8 for AD; SERPINE2 for PD; EGF, TPP1 and ATXN3 for ALS; and CTSH, IL7R, PARP1 and IDUA for MS). We also observed that 11 protein–disease associations reached statistical significance only when the lead *cis* pQTL from the SomaScan platform was used but not when the lead *cis* pQTL from the Olink platform was used (CTSH, LGALS3, PARP1 and LILRB1 for AD; IDUA for PD; CD200, INHBC, THSD1 and AHSG for MS; and IDUA and INHBC for ALS; Supplementary Fig. 3).

### Sensitivity analysis for Alzheimer’s disease

The GWAS by Bellenguez *et al*.^9^ used proxy cases for AD, where possible cases identified through self-reported family history of dementia were included; these contributed 42% of AD cases, with the balance being physician-diagnosed cases of AD.^9^ As a sensitivity analysis, we used the GWAS by Kunkle *et al*.^44^ to validate that the proteins associated with AD are specific for AD, because it is the latest GWAS for AD that does not include proxy cases (Supplementary Table 3).^44^ Out of 78 protein–AD associations, 23 (28%) were associated with AD in the MR analysis using the GWAS by Kunkle *et al*.^44^ at 5% FDR (Supplementary Fig. 4). All the validated associations were directionally consistent across the two GWAS for AD. Among the non-validated associations, four associations (ARHGAP45, EPHX2, PARP1 and PLOD2) were not directionally consistent. The non-validation of some of the proteins could be explained either by the smaller sample size of the study by Kunkle *et al*.^44^ (i.e. loss of statistical power) or by the misclassification of other causes of dementia as AD in the study by Bellenguez *et al*.^9^

### Sensitivity analysis for reverse causation

To explore the potential for reverse causation, we performed a bi-directional MR analysis, which examines whether the genetic liability to the outcome is associated with the exposure of interest. For this reason, we performed clumping to identify independent genome-wide significant variants (*P* < 5.00 × 10^−8^) from the GWAS for AD, PD, ALS and MS, for use as IVs modelling the genetic liability to these diseases. Using a random-effects inverse-variance weighted model, we found that genetically predicted risk for AD was associated with plasma protein abundance of APOE and CEACAM19, genetically predicted risk for ALS was associated with DTX3, and genetically predicted risk for MS was associated with plasma protein abundance of CD58, JUND, CD200, PGLYRP1, TNFRSF1A, LMAN2, INHBC, DKKL1, AHSG and KLRB1 (5% FDR; Supplementary Table 4).

### Co-localization between plasma protein abundance and neurodegenerative diseases

To examine whether confounding by LD can explain the observed associations, we performed co-localization of the association signals for protein and disease, assuming a single causal variant at each gene locus (Supplementary Table 5).^25^ Among the 169 protein–disease pairs, 61 (36%) had strong evidence of co-localization (posterior probability > 0.80), and 35 additional protein–disease pairs (21%) showed moderate evidence of co-localization (0.60 < posterior probability < 0.80). CR1 co-localized with both AD and MS. Eleven protein–disease pairs showed strong evidence of co-localization in both Olink and SomaScan platforms (C1R, C1S, CR1, GRN, PILRA, SIGLEC9, SIRPA for AD; and CR1, DKKL1, TNFRSF1A and WARS for MS).

The 61 protein–disease pairs with strong evidence of co-localization correspond to 50 unique protein–disease associations and 49 unique proteins, which were prioritized for further analyses (Tables 2 and 3). Twenty-three of these 50 protein–disease pairs (48%) represent previously unreported genetic associations, because they did not reach genome-wide significance in the disease GWAS, nor have they been otherwise previously reported in the GWAS Catalog (Tables 2 and 3).^28^ Although *PRSS8* has not been previously reported as a genetic locus involved in AD, the *cis* pQTL for PRSS8 is a previously identified genome-wide significant association for AD, which is located within a different nearby gene (*BCKDK*) related to AD.^9^

### Association of plasma mRNA abundance with neurodegenerative diseases

We performed an additional two-sample MR analysis to investigate the association of the plasma abundance of mRNA encoding the proteins associated with risk of neurodegenerative diseases using the lead *cis* eQTL reported by the eQTLGen consortium.^46^ We tested 7259 plasma mRNA abundance–disease associations, and 90 (1.2%) were statistically significant at 5% FDR, which corresponds to *P* < 6.2 × 10^−4^. Among these 90 associations, 43 (32.5%) were also statistically significant using the lead plasma *cis* pQTL (Supplementary Table 6 and Supplementary Fig. 5). When we compared the associations using plasma pQTLs and plasma eQTLs (Supplementary Fig. 6), we found inconsistencies in the direction of effect in six associations using the Olink platform (CEACAM19 and TREML2 with AD; TPP1 with ALS; and CD58, IL2RA and PTPRC with MS) and two associations using the SomaScan platform (PILRA with AD; and IL2RA with MS).

Thirteen of 43 associations (30%) also showed evidence of co-localization across the locus between mRNA abundance and disease risk (Supplementary Table 7). Ten proteins showed co-localization with a neurological disease using both plasma protein abundance and plasma mRNA abundance (SIRPA, CTSH and CD33 with AD; and ASF1A, FCRL3, VEGFB, TYMP, PVALB, LMAN2 and CD5 with MS). The remaining three proteins (ACE with AD; SIGLEC9 with ALS; and PLEK with MS) showed evidence of co-localization with plasma mRNA abundance but not with plasma protein abundance. There is strong evidence for distinct causal variants between ACE protein abundance and AD (posterior probability = 1.00), and moderate evidence for shared causal variants of PLEK protein abundance with MS and SIGLEC9 with ALS (posterior probability = 0.74 and 0.70, respectively). Discrepancies in the MR and co-localization analyses are expected because of the uncoupling of gene and protein expression, which is a frequently described phenomenon attributed to differential translation, protein degradation, contextual confounders such as time and developmental state, or protein-level buffering.^61,62^

### Association of brain mRNA abundance with neurodegenerative diseases

As an additional step to aid interpretation of our findings, we used data on genetic associations with mRNA abundance in four brain regions (i.e. cortex, basal ganglia, hippocampus and cerebellum) and the spinal cord, as provided by the MetaBrain consortium.^47^ For each protein measured through Olink or SomaScan platforms, we selected the lead *cis* eQTL per tissue as an IV, testing a total of 16 991 mRNA abundance–disease associations (8803 in cortex, 5881 in cerebellum, 1013 in basal ganglia, 710 in hippocampus and 584 in spinal cord), of which 188 (1.1%) were statistically significant at 5% FDR, which corresponds to *P* < 5.5 × 10^−4^. Of 188 associations, 96 (51%) were also statistically significant using the plasma lead *cis* pQTL (Supplementary Table 8). When we compared these associations with the associations using the lead plasma *cis* pQTL, we found that 74 (56 with Olink and 18 with SomaScan) were consistent in the direction of effect, whereas 22 (15 with Olink and 7 with SomaScan) were directionally inconsistent (Supplementary Figs 7–12).

Thirty-four of these 96 associations (35%) showed strong evidence of co-localization (Supplementary Table 9). Two proteins (CR1 with AD in basal ganglia, cortex and hippocampus; and GRN with AD in cerebellum, cortex and hippocampus) were found to have evidence for co-localization with disease risk from mRNA abundance in three brain regions, and a further six proteins (ACE, CD33, CTSH and SIRPA with AD in cerebellum and cortex; LRRC37A2 with AD in cortex and hippocampus; and PVR with MS in cerebellum and cortex) have support for co-localization with mRNA abundance in two brain regions. Of note is that six proteins did not show evidence of co-localization with plasma protein abundance, but they co-localized with mRNA abundance in one or more brain regions (UBASH3B, NSF, LRRC37A2, BIN1 and ACE with AD; and PTPRJ with MS). Discrepancies in the MR and co-localization analyses are expected, because plasma pQTLs show the least concordance and co-localization with brain eQTLs, which could be attributed to factors affecting access to the circulation, such as the blood–brain barrier (BBB).^26^

### Association of plasma protein abundance with brain imaging traits

To assess whether the identified proteins might have an impact on brain structure, we examined the association of *cis* pQTLs for the proteins measured through either Olink or SomaScan platforms with 12 brain-imaging traits (i.e. intracranial brain volume, mean cortical thickness and surface area, eight subcortical brain volumes, and white matter hyper-intensities). Out of 41 738 tested associations, 3272 (7.8%) were nominally significant at *P* < 0.05, but only 101 (0.2%) were significant at 5% FDR (equivalent to *P* < 1.2 × 10^−4^). Out of 101 statistically significant associations, we focused on seven associations that represented proteins associated with at least one neurodegenerative disease with additional support from co-localization (Supplementary Table 10). All these latter associations were supported by co-localization with a brain imaging trait (Supplementary Table 11). Plasma APOE abundance was associated with hippocampal volume, amygdala volume, nucleus accumbens volume and white matter hyper-intensities. Plasma PILRA and PILRB abundance was associated with caudate nucleus volume. PILRA co-localized with caudate nucleus volume using a *cis* pQTL from either Olink or SomaScan platforms.

### Cumulative evidence from *cis* protein and expression quantitative trait loci

**Figure 5:**
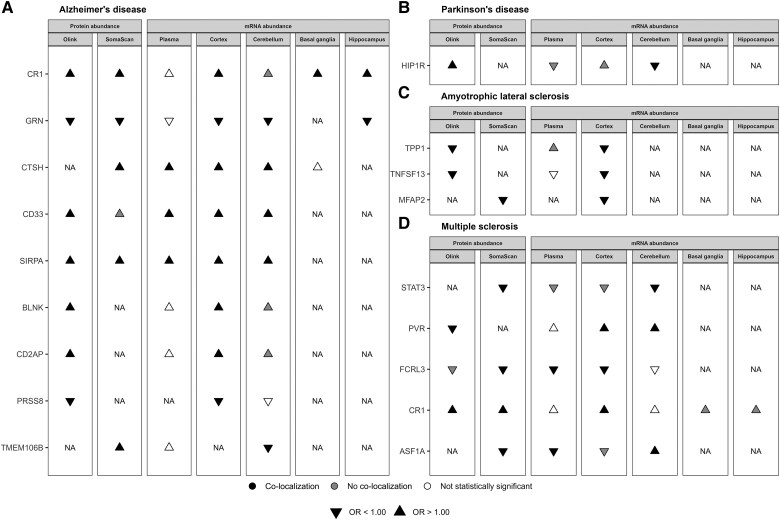
**Summary of cumulative evidence using plasma *cis* protein quantitative trait loci and plasma or brain *cis* expression quantitative trait loci as instrumental variables for proteins associated with neurodegenerative diseases.** This figure shows the 18 proteins that demonstrated evidence of association and co-localization when we used plasma protein quantitative trait loci (pQTLs) and expression quantitative trait loci (eQTLs) in at least one brain region. The remaining proteins are presented in Supplementary Fig. 13. **A**–**D** each correspond to one neurodegenerative disease: (**A**) Alzheimer’s disease; (**B**) Parkinson’s disease; (**C**) amyotrophic lateral sclerosis; and (**D**) multiple sclerosis. When the Mendelian randomization analysis did not show a statistically significant effect, co-localization was not performed. NA (Not Available) indicates that no *cis* pQTL or *cis* eQTL was available for a particular protein.

Our study has combined evidence from MR and co-localization to identify potentially causal relationships between proteins and neurodegenerative diseases using genetic associations with plasma protein abundance, plasma mRNA abundance, and brain and spinal cord mRNA abundance. On the basis of the overall evidence for association with neurological diseases, we defined three categories of evidence (Fig. 5 and Supplementary Fig. 13). In Category 1, we identified 18 proteins that showed evidence of association and co-localization when we used a plasma pQTL and an eQTL in at least one brain region. These proteins are TMEM106B, SIRPA, PRSS8, GRN, CTSH, CR1, CD33, CD2AP and BLNK for AD; HIP1R for PD; TPP1, MFAP2 and TNFSF13 for ALS; and STAT3, PVR, FCRL3, CR1 and ASF1A for MS (Fig. 5). In Category 2, we identified five proteins that showed evidence of association and co-localization when we used a plasma pQTL and plasma eQTL (but not studied or detected in any brain region). In Category 3, we identified 26 proteins that showed association and co-localization only when we considered a plasma pQTL but not a plasma or brain eQTL.

### Downstream analyses

To gain a better understanding of the biological implications of our findings, we examined whether the prioritized proteins show high specificity in particular tissues and single cells, and we assessed their presence in distinct gene expression clusters, using data derived from the Human Protein Atlas (Supplementary Table 12 and Supplementary Fig. 14).^59^ Although the majority of the proteins presented low tissue specificity, multiple proteins were present in the tissue expression clusters related to liver, lymphoid tissue and spleen. Likewise, although most proteins showed low brain regional specificity, several proteins were present in the brain expression clusters related to astrocytes, macrophages and microglia. At the single-cell level, the majority of the proteins showed high specificity in cells related to the immune system.

To assess whether the prioritized proteins indicate specific biological mechanisms underlying neurodegenerative diseases, we performed an enrichment analysis. Our analysis highlighted the involvement of the innate and adaptive immune system, and the role of lysosomes in AD. Likewise, proteins associated with MS were enriched for immune-related processes (Supplementary Table 13). In contrast, no enrichment was observed for the proteins associated with PD and ALS.

## Discussion

This study has systematically assessed the associations of >2700 proteins with four neurodegenerative diseases using summary statistics from large-scale proteogenomic data and the latest GWAS for disease risk. We identified 50 unique associations between plasma protein abundance and neurodegenerative diseases with support from MR and evidence of co-localization. Twenty-seven of these associations are known disease loci reported in GWAS, including *APOE*, *MME*, *CD2AP*, *CD33* and *IL34* for AD, and *CD40*, *CD58*, *EVI5*, *IL7R* and *STAT3* for MS, and 23 associations represent previously unreported genetic associations with neurodegenerative diseases.

### Proteins related to Alzheimer's disease

Accumulation of β-amyloid is a key pathological feature for AD. APOE is a protein directly involved in the regulation of the β-amyloid aggregation and clearance in the brain.^63^ CD2AP actively participates in the metabolism of β-amyloid, and knockout of *CD2AP* results in endosomal accumulation of β-amyloid in animal models.^64^ MME is also another important enzyme of β-amyloid degradation.^65^

Our findings highlight the role of microglia, which is a cell type equivalent to peripheral macrophages in the brain responsible for the clearance of β-amyloid peptides.^66^ Of note is that our analysis contributes three newly reported AD loci related to microglial function (*SIRPA*, *SIGLEC9* and *PRSS8*). *CD33* and *IL34* are expressed in microglia and inhibit the microglial uptake of β-amyloid and, therefore, influence the accumulation of amyloid plaque.^67,68^ Anti-CD33 antibodies are used for the treatment of acute myeloid leukaemia and have previously been suggested for drug repurposing for AD.^69^ SIGLEC9 participates in the immune response to several bacterial pathogens by reducing bacterial dissemination into the brain and exerts neuro-protective effects by suppressing inflammatory responses to the brain.^70^ SIRPA regulates microglial phagocytosis and the transmigration of monocytes across the BBB and participates in the pathogenesis of neurodegeneration in preclinical models.^71,72^ PRSS8 modulates Toll-like receptor 4, which is a receptor in the membrane of microglia and contributes to microglial activation and phagocytosis of β-amyloid.^73,74^ The complement system regulates microglial function and neuro-inflammation,^75^ and we identified one known locus (*CR1*) and two newly identified loci (*C1R* and *C1S*) for AD.

Herpes simplex virus-1 (HSV-1) has been linked with neurodegeneration and cognitive defects in mouse animal models.^76,77^ HSV-1 binds to PILRA, a protein associated with AD, to infect cells. PILRA is a cell surface inhibitory receptor expressed on innate immune cells, including microglia.^78,79^ In our analysis, PILRA was associated with caudate nucleus atrophy, which has been previously observed in other neurodegenerative diseases, including frontotemporal dementia, PD and Huntington’s disease.^80-82^ This finding could indicate that HSV-1 participates in the pathogenesis of AD by affecting caudate nucleus.

There is an increasing amount of evidence supporting the role in neurodegenerative diseases of lysosomes,^83^ which play an important role in phagocytic cells, including microglia.^84^ Our analysis identified three known (*GRN*, *TMEM106B* and *CTSH*) and one novel (*CLN5*) AD-associated loci related to lysosomal functions. GRN protects against β-amyloid deposition and toxicity in AD mouse models, and its deficiency has been linked to neural circuit development and maintenance, stress response and innate immunity.^84,85^ TMEM106B has previously been linked to frontotemporal dementia, and there is evidence of its interaction with GRN; both of them are considered crucial markers of brain ageing.^86^ Moreover, genetic deficiency of either CLN5 or GRN is responsible for an inherited lysosomal disease.^87,88^ Loss of CLN5 leads to deficits in neurodevelopment in mice models.^87^ CTSH belongs to the cathepsin superfamily, which is a large group of proteases located in the lysosomes.^89,90^ Knockout of *CTSH* affects the phagocytosis of amyloid-β in microglial cells.^90^

Our analysis found one known protein (BLNK) and two additional newly identified proteins (PLOD2 and ZBTB18) potentially participating in the pathogenesis of AD through other pathways. *PLOD2* is overexpressed in fibroblasts, strengthening the current evidence for a potential role for fibroblasts in the pathogenesis of AD through remodelling of the extracellular matrix alongside amyloid plaques.^91^ ZBTB18 is an essential transcription factor for embryonic cerebral cortex development^92^; it has been identified as a contributing factor to the 1q43q44 microdeletion syndrome, which is characterized by variable intellectual disability and brain malformations.^93^ BLNK is involved in B-cell receptor signalling; although the role of B cells in AD is not well understood, targeting B cells has been suggested to be beneficial for AD patients by delaying disease progression.^94^

### Proteins related to Parkinson's disease and amyotrophic lateral sclerosis

Although the GWAS for PD and ALS have relatively small sample sizes, our study was nevertheless able to identify one novel locus for PD (*CTF1*) and three novel loci for ALS (*TPP1*, *TNFSF13* and *MFAP2*). CTF1 is a neurotrophic factor in the interleukin-6 cytokine family. Pro-inflammatory cytokines, including interleukin-6, have previously been associated with PD.^95^ It has also been shown in a mouse model that *CTF1* transfection and expression is neuroprotective and slows progression of spinal muscular atrophy.^96^ One of the aetiologies of ALS is mis-localization of TDP-43 to mitochondria, causing neurotoxicity.^97^ TPP1 is a lysosomal enzyme, and loss-of-function mutations in the gene are causally linked to a familial lysosomal disorder, in which *TPP1* loss affects regulation of axonal mitochondrial transport.^98^ Also, loss of TPP1 activity results in progressive neurological phenotypes, including ataxia and increased motor deficiency.^99^  *TNFSF13* is expressed in astrocytes and regulates neuro-inflammatory responses.^100^ Reactive astrocytes have neurotoxic properties and are involved in the pathogenesis of ALS.^101^ Although MFAP2 has not previously been linked with ALS, there are potential mechanisms that can explain this association. MFAP2 is essential in maintaining vessel wall integrity, and its dysfunction leads to BBB disruption.^102^ MFAP2 is also secreted to the extracellular matrix, and its composition in the brain affects the integrity of neurons.^103,104^

### Proteins related to multiple sclerosis

Experimental autoimmune encephalomyelitis is an animal model for MS. Nine of the MS-associated proteins, six known (CD5, CD40, IL7R, STAT3, TNFRSF1A and TYMP) and three newly associated with MS (PARP1, PVALB and VEGFB), are involved in the pathogenesis of experimental autoimmune encephalomyelitis.^105-113^ This observation strengthens the validity of our findings.

The innate immune system participates in pathogen removal and regulates the response of the adaptive immune system,^114^ including the response to Epstein–Barr virus infection, which is a pathogen associated with MS.^115^  *PVR*, a known locus for MS, encodes the polio virus receptor, which is involved in the immune response to Epstein–Barr virus. Increased expression of *PVR* downregulates the expression of microRNAs produced by Epstein–Barr virus,^116^ which potentially explains the apparent protective effect of higher plasma levels of PVR in our analysis. WARS, a protein newly associated with MS, is an aminoacyl-tRNA synthetase with a role as an innate immune activator in the extracellular space, acting as a primary defence system against infections, especially antiviral immunity.^117,118^ Moreover, a newly identified MS protein, PARP1, is involved in the nuclear factor-κB signalling pathway,^119^ which is activated as a response to infectious antigens, including Epstein–Barr virus,^120^ and is an important pathway for the activation of macrophages and other innate immune cells.^119^ The newly identified association of CR1 with MS risk indicates a role of complement, which is an important innate immune defence against infection, as has been suggested recently.^121^ SLAMF1 and AHSG participate in Toll-like receptor 4 signalling, which activates macrophages against bacterial pathogens.^122,123^ This observation potentially provides support to the hygiene hypothesis for the development of MS.^124^

The adaptive immune system consists of B cells and T cells, which are activated by innate immune cells. IL7R has a role in T- and B-cell differentiation, and its plasma levels are associated with elevated risk of MS,^125^ but experimental IL7R inhibitors have not been successful in treating MS.^126^ CD5 and CD58 are also involved in B- and T-cell differentiation, whose activation has a role in autoimmunity.^127,128^ CD40 and its ligand form a complex that has a central role in the regulation of both humoral and cell-mediated immunity. Blockade of CD40L is effective in ameliorating experimental autoimmune conditions, and it has also been suggested as a potential therapeutic strategy for MS.^129^ Also, *FCRL3* is mainly expressed in B cells, and it has been linked to multiple autoimmune conditions.^130^

Demyelinating lesions in white and grey matter are the histopathological landmarks of MS, which are infiltrated by cells of the innate and adaptive immune system,^114^ whereas oligodendrocytes are responsible for the myelination process.^131^ PARP1, a newly associated protein for MS, is a driver for oligodendroglial development and myelination,^132^ and PARP1 inhibitors have been suggested as a potential therapy for MS,^133^ in line with our finding that elevated plasma PARP1 is associated with increased MS risk. STAT3, a known protein for MS, is important for myelin repair, and pharmacological blockade of STAT3 activation with JAK2 inhibitors inhibits survival and differentiation of oligodendrocyte precursor cells.^134^ Another known protein for MS, TNFRSF1A, is involved in the TNF receptor-associated periodic syndrome, which is characterized by inflammatory demyelination. There is evidence that anti-TNFα therapies can result in new episodes of inflammation in MS patients.^135^

The BBB protects the CNS parenchyma from harmful circulating molecules and pathogens,^136^ and altered BBB function is believed to be an important early stage in MS pathology. Several identified proteins have potential roles in the BBB, including TYMP, a key astrocyte-derived permeability factor promoting BBB breakdown,^106^ CD40, which influences the permeability of the BBB,^137^ and VEGFB, a newly identified MS-associated protein that is a member of the vascular growth factor family, again involved in the permeability of the BBB.^138^

The node of Ranvier on white matter demyelinated axons is profoundly altered or disrupted in patients with MS,^139,140^ and two newly identified proteins (NCS1 and CDH15) are involved in its function. NCS1 is involved in the regulation of intracellular calcium signalling and is identified in the nodes of Ranvier. NCS1 also participates in the pathogenesis of chemotherapy-induced peripheral neuropathy.^141^ A member of the cadherin protein family, CDH15, participates in the function of the node of Ranvier and has previously been associated with chronic inflammatory demyelinating polyneuropathy, a demyelinating disease of the peripheral nervous system.^142^ Moreover, lesions in the dorsal root ganglion are identified in experimental autoimmune encephalomyelitis,^143,144^ and FLRT3, a newly identified protein related to MS, is overexpressed in the dorsal root ganglion and has been associated with neuropathic pain in animal models.^145^

Three further newly identified MS-associated loci (*PVALB*, *TST* and *ASF1A*) potentially indicate additional molecular pathways contributing to MS. PVALB is specifically expressed by GABAergic interneurons and has been suggested as a potential MS-specific marker of grey matter neurodegeneration.^146^ TST is an enzyme involved in mitochondrial sulphur and selenium metabolism,^147^ and it has been shown that exposure to oxidative stress owing to mitochondrial dysfunction contributes to the chronic demyelination.^148^ ASF1A is a histone chaperone that has been implicated in neuro-inflammation and neurodegeneration processes through activation of microglia.^149,150^

### Comparison of Mendelian randomization studies and observational studies

Observational studies have examined the association between the plasma proteome and risk of AD.^151-153^ We observed that there is no agreement between the proteins in our study and the proteins identified from the observational analyses. Some proteins do not have a *cis* pQTL, and they could not be tested in our analysis. There are also additional reasons that could lead to differences between observational studies and MR studies that should be acknowledged. First, the observational analyses adjusted for APOE ε4 status, and there is evidence that APOE ε4 status modifies proteomic signatures in AD.^154^ Second, effect estimates based on MR assume a lifelong exposure to altered protein levels from birth.^155^ In contrast, observational analyses are based on the human proteome measured at a single time point in adult life. Third, a *cis* pQTL for a particular protein can have pleiotropic effects, acting as *trans* pQTL for other proteins. Fourth, statistical power of an MR analysis depends on the explained variability of the protein abundance from the *cis* pQTL.^156^ Therefore, using a lead *cis* pQTL with a low explained variability might lead to an underpowered MR analysis. Fifth, results from observational analyses are sensitive to the selection of covariates for adjustment.^157^ For example, by adjusting for a covariate that mediates the effect of a protein on risk of AD, the causal pathway is blocked, hindering the identification of an association in an observational analysis.^158^ Also, adjusting for a covariate that is a collider introduces collider bias that leads to spurious associations. Currently, there are no large prospective studies examining the role of the human proteome in the development of PD, ALS or MS.

### Plasma and tissue-specific proteomic effects

The identification of proteins with roles in many of the biological processes relevant to neurodegenerative diseases supports the idea that targeting such proteins might form the basis of future drug development. However, it seems likely that abundance of these proteins in plasma is not directly relevant to disease pathology, and that therapies will need to be targeted to the relevant tissue or cell type. Although a drug might modify levels of the identified proteins in plasma, we cannot assume that it would cross the BBB for brain-targeting drugs.^159^ Nevertheless, although our results are based primarily on proteins measured in plasma, it is plausible that the same genetic factors have similar effects on protein levels in more relevant tissues, and that our results reflect similarities in processes such as macrophage activity, lysosomal activity and β-amyloid metabolism in blood and brain. For example, in the pathogenesis of MS, the activation of the innate and adaptive immune system occurs initially in the periphery and is then transferred to the CNS.^114^ In particular, *cis* pQTLs, particularly those directly impacting protein-coding sequences, will frequently have similar effects across diverse tissues.^160^

### Limitations

There are several limitations to the present study. First, our analyses are underpowered for PD, because full summary statistics including 23andMe are not publicly available, greatly reducing the sample size in the available data. Second, GWAS for the neurological diseases primarily assess risk for disease and not disease progression. Therefore, the identified proteins can be considered as potential biomarkers for prediction or diagnosis of the diseases or drug targets for disease prevention but not necessarily for disease progression.^161^ Third, our study is constrained by the number of proteins that can be analysed using Olink and SomaScan platforms, which cover only a fraction of the entire proteome. Fourth, our co-localization analysis assumed the presence of a single causal genetic variant per genetic locus. Absence of co-localization could be observed in the case of violation of this assumption, where more than one causal genetic variant exists in a particular genetic locus for a particular trait.^25^ Fifth, sample overlap can bias the results of an MR analysis.^162^ For the MR effect estimates using pQTLs from the SomaScan platform, there is no sample overlap for any of the traits of interest. For the MR analyses on ALS and MS, there is no inclusion of participants from the UK Biobank or deCODE Health Study in the respective GWAS. For the MR analyses on AD and PD using *cis* pQTLs from Olink platform, there is a sample overlap of <5%. Owing to the minimal sample overlap, we expect that our results will not be impacted substantially by this.

## Conclusion

We have presented a comprehensive analysis of associations of the plasma proteome with neurodegenerative diseases by considering proteins measured through either Olink or SomaScan platforms. We identified multiple proteins with a potential causal role in neurodegenerative diseases. The newly identified proteins for AD are involved in the immune response to bacterial pathogens, complement system, transmigration of monocytes across the BBB, Toll-like receptor 4 signalling, lysosomal function and fibroblasts. The newly identified proteins for MS are involved in the innate immune system, complement, microglia, oligodendrocytes, permeability of the BBB, GABAergic interneurons and the function of the node of Ranvier and dorsal root ganglion. Our analysis covered only a modest proportion of the human proteome and was limited to proteins measured in plasma; therefore, further expansion of the multiplexed antibody-based and aptamer-based assays and conducting large-scale assays in more directly relevant tissues will offer additional insights into the role of protein abundance in the development of neurodegenerative diseases. Moreover, better characterization of the protein isoforms targeted by these complementary proteomics platforms will offer additional insights into the biological interpretation of the findings.

## Supplementary Material

awaf018_Supplementary_Data

## Data Availability

Descriptive characteristics of the data sources used in this study are shown in Supplementary Table 1. The *cis* pQTLs that were used as IVs are publicly available in the relevant publications.^26,27^ Summary statistics for the GWAS on AD, PD, ALS and MS are available through GWAS Catalog (https://www.ebi.ac.uk/gwas/). Summary statistics for the GWAS on brain volume traits are available upon request from the ENIGMA consortium (https://enigma.ini.usc.edu/). Summary statistics for the GWAS on white matter hyper-intensities are publicly available through the Cerebrovascular Disease Knowledge Portal (https://cd.hugeamp.org/). Summary statistics for the *cis* region of gene expression in plasma and brain regions are publicly available through the eQTLGen (https://www.eqtlgen.org/) and the MetaBrain (https://www.metabrain.nl/) consortia, respectively.
