# Supplementary material for: Mendelian randomization identifies proteins involved in neurodegenerative diseases
Source: Brain. 2025 Mar 3;148(7):2412–28. doi: 10.1093/brain/awaf018 (PMC12233555; doi:10.1093/brain/awaf018)
Supplement: awaf018_Supplementary_Data [file awaf018_supplementary_data.zip › awaf018_Supplementary_Figures.pdf]

SUPPLEMENTARY MATERIAL

Mendelian randomization identifies proteins involved in neurodegenerative diseases

Lazaros Belbasis[1], Sam Morris[1], Cornelia van Duijn[1], Derrick Bennett[1], Robin Walters[1]

Author affiliations:

1 Nuffield Department of Population Health, University of Oxford, OX3 7LF Oxford, UK

Supplementary Figures

Supplementary Figure S1. Plasma proteins associated with more than one neurodegenerative diseases using two-sample MR.

Supplementary Figure S2. Overlap of the statistically significant protein-disease associations using plasma *cis* pQTLs and a Mendelian randomisation approach across four neurodegenerative diseases.

Supplementary Figure S3. Comparison of the Z-statistics for the association between plasma protein abundance and neurodegenerative diseases across the Olink and SomaScan platform.

Supplementary Figure S4. Comparison of the MR effect of plasma protein abundance on Alzheimer's disease using two different genome-wide association studies for Alzheimer's disease.

Supplementary Figure S5. Overlap of the statistically significant protein-disease associations using plasma *cis* pQTLs, plasma *cis* eQTLs and a Mendelian randomisation approach across four neurodegenerative diseases.

Supplementary Figure S6. Comparison of Z-statistics for the association of plasma protein abundance and plasma mRNA abundance with neurodegenerative diseases.

Supplementary Figure S7. Overlap of the statistically significant protein-disease associations using plasma *cis* pQTLs, cortex *cis* eQTLs and a Mendelian randomisation approach across four neurodegenerative diseases.

Supplementary Figure S8. Overlap of the statistically significant protein-disease associations using plasma *cis* pQTLs, cerebellum *cis* eQTLs and a Mendelian randomisation approach across four neurodegenerative diseases.

Supplementary Figure S9. Comparison of the Z-statistics for the association of plasma protein abundance and mRNA abundance in brain with Alzheimer's disease.

Supplementary Figure S10. Comparison of the Z-statistics for the association of plasma protein abundance and mRNA abundance in brain with Parkinson's disease.

Supplementary Figure S11. Comparison of the Z-statistics for the association of plasma protein abundance and mRNA abundance in brain with amyotrophic lateral sclerosis.

Supplementary Figure S12. Comparison of the Z-statistics for the association of plasma protein abundance and mRNA abundance in brain with multiple sclerosis.

Supplementary Figure S13. Summary of cumulative evidence using plasma *cis* pQTLs and plasma or brain *cis* eQTLs as instrumental variables.

Supplementary Figure S14. Tissue, brain, and single cell specificity and expression clusters for proteins associated with neurodegenerative diseases.

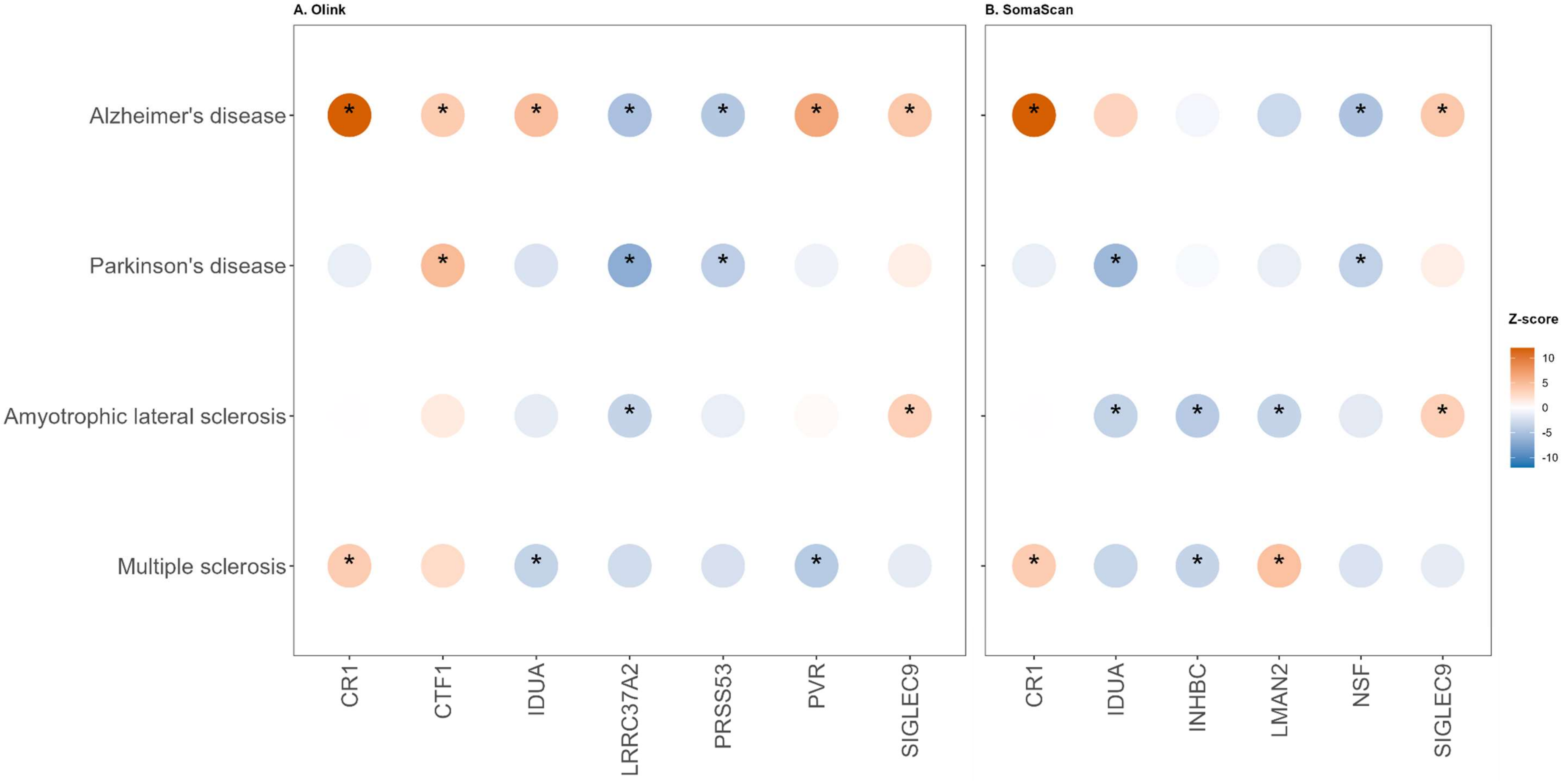

Supplementary Figure S1. Plasma proteins associated with more than one neurodegenerative diseases using two-sample MR. Seven plasma proteins measured through the Olink platform (CR1, CTF1, IDUA, LRRC37A2, PRSS53, PVR, and SIGLEC9) and six plasma proteins measured through the SomaScan platform (CR1, IDUA, INHBC, LMAN2, NSF, and SIGLEC9) were associated with more than one neurodegenerative disease at 5% false discovery rate.

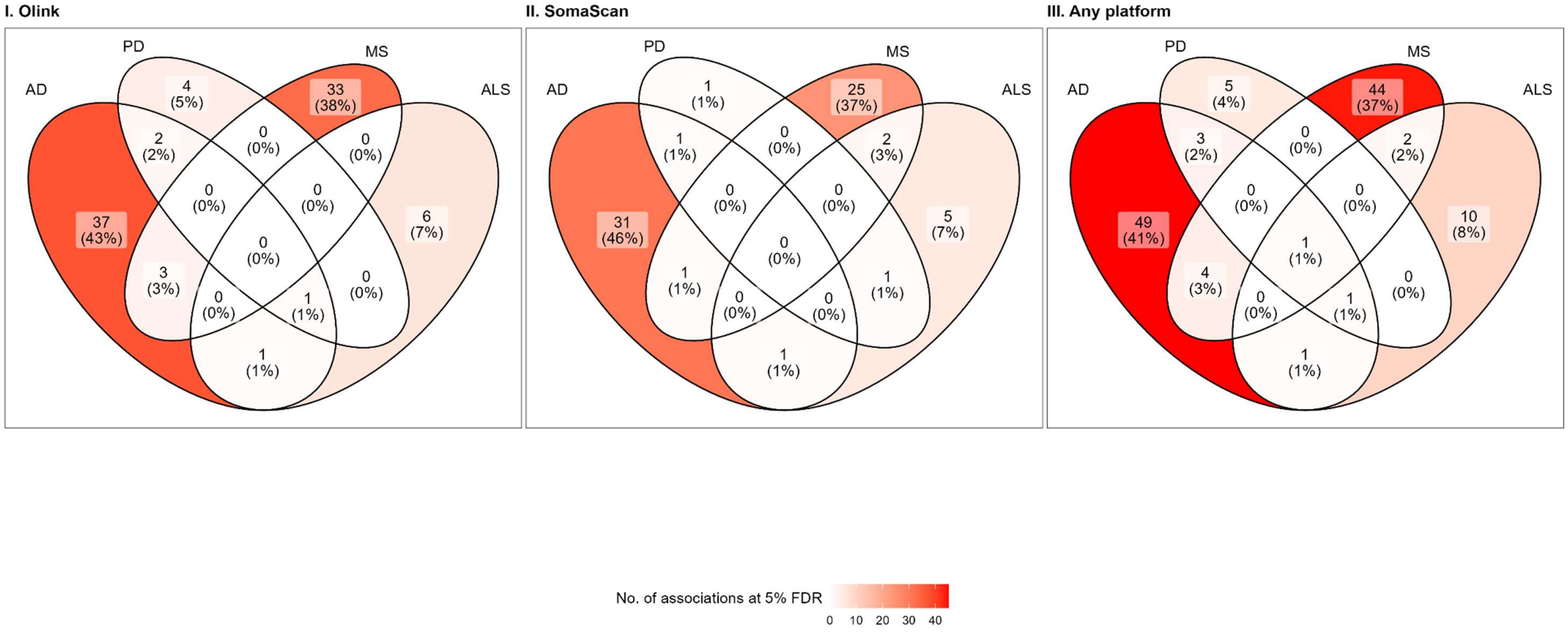

Supplementary Figure S2. Overlap of the statistically significant protein-disease associations using plasma *cis* pQTLs and a Mendelian randomisation approach across four neurodegenerative diseases. The Venn diagrams show the number and percentage of proteins significantly associated with each disease or combination of diseases, based on a 5% false discovery rate (FDR) threshold. The three panels represent associations identified using (I) the Olink platform, (II) the SomaScan platform, and (III) any platform. The intensity of the red shading reflects

the number of significant associations, with darker shades representing a greater number of associations. AD, Alzheimer’s Disease; PD, Parkinson’s Disease; ALS, Amyotrophic Lateral Sclerosis; MS, Multiple Sclerosis.

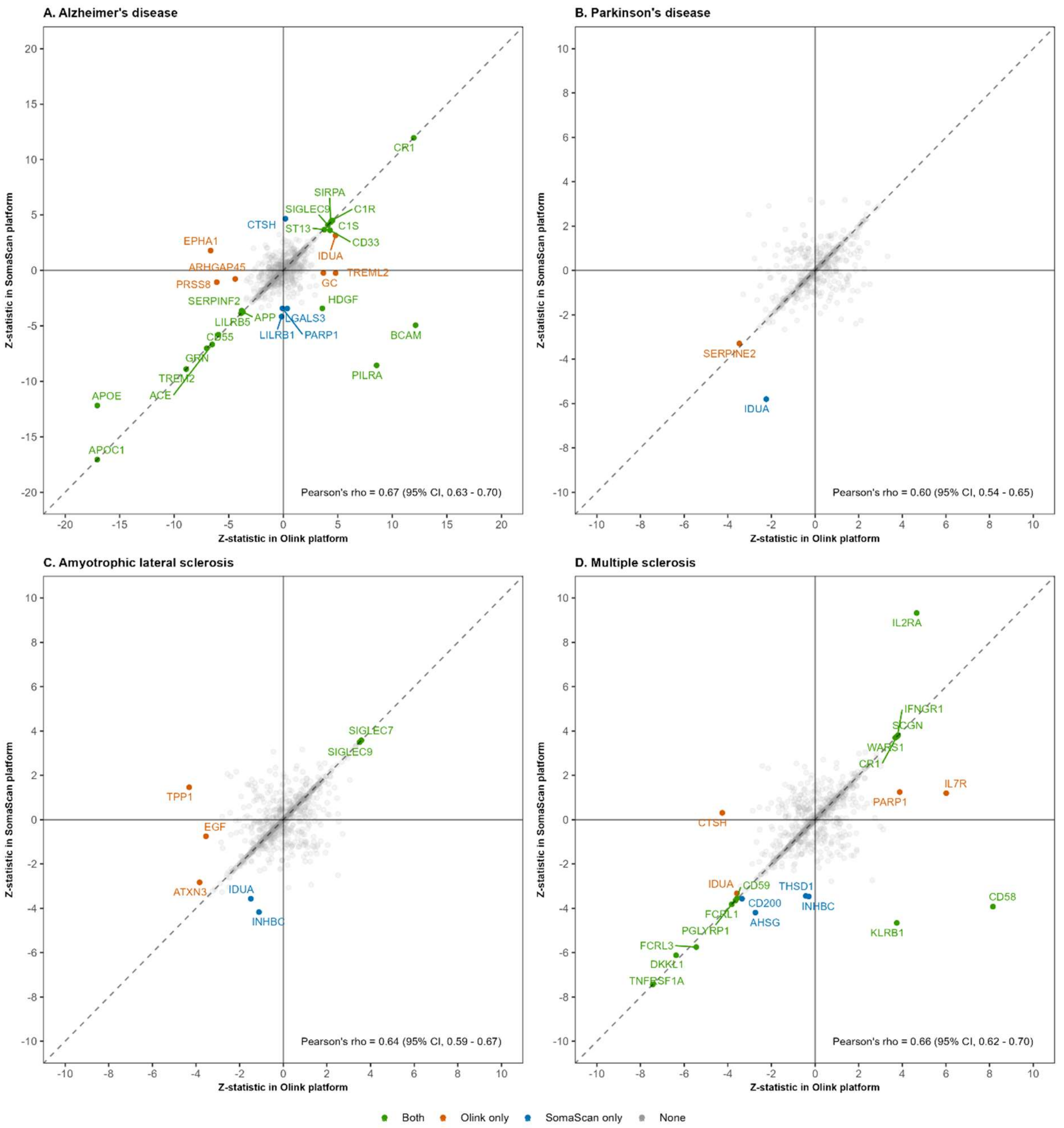

Supplementary Figure S3. Comparison of the Z-statistics for the association between plasma protein abundance and neurodegenerative diseases across the Olink and SomaScan platform. Each panel corresponds to a specific neurodegenerative disease: (A) Alzheimer's Disease, (B) Parkinson's Disease, (C) Amyotrophic Lateral Sclerosis, and (D) Multiple Sclerosis. Proteins identified as statistically significant based on the lead *cis* pQTL from either Olink or SomaScan are annotated and color-coded. The diagonal dashed line indicates perfect correlation, and Pearson's correlation coefficient (rho) with a 95% confidence interval (CI) is reported for each disease, reflecting the overall agreement between platforms.

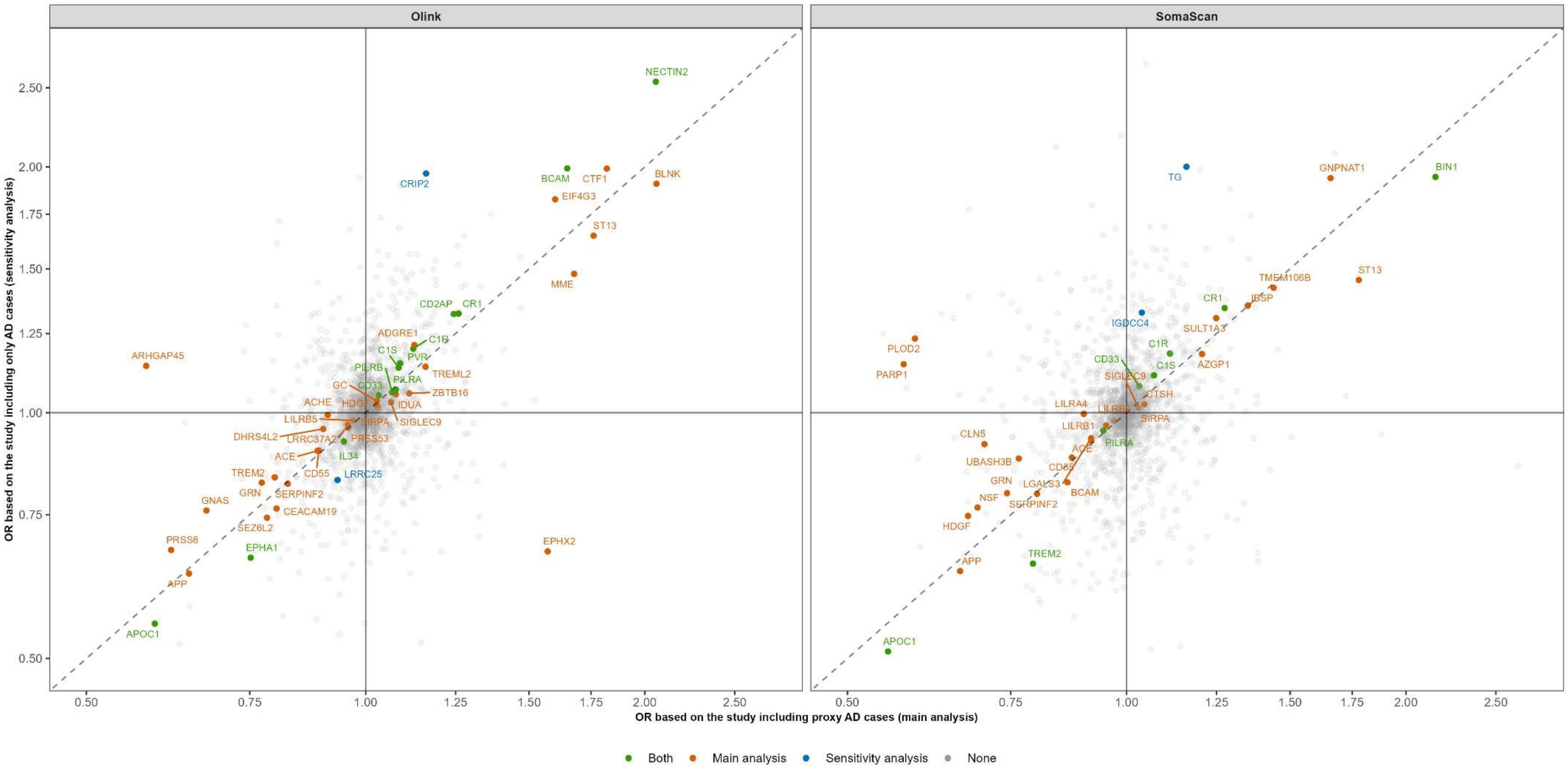

Supplementary Figure S4. Comparison of the MR effect of plasma protein abundance on Alzheimer's disease using two different genome-wide association studies for Alzheimer's disease. The x-axis represents the odds ratios (ORs) from the GWAS incorporating both physician-diagnosed AD cases and proxy AD cases (Bellenguez et al.), while the y-axis represents the ORs from the GWAS based solely on physician-

diagnosed AD cases (Kunkle et al.). Proteins that are statistically significant in either GWAS are annotated in the plots. Colour coding reflects the statistical significance based on a 5% false discovery rate (FDR) threshold.

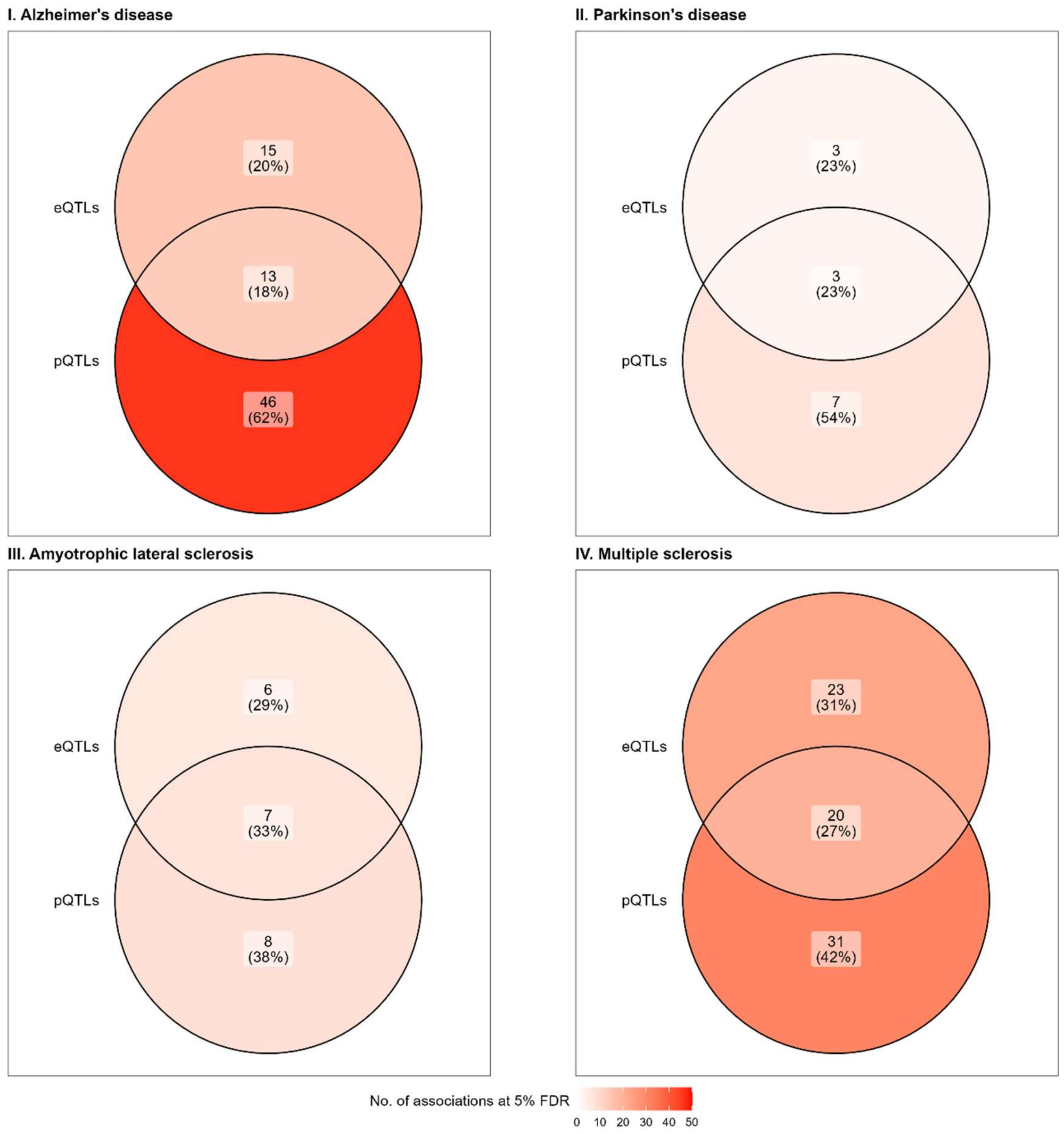

Supplementary Figure S5. Overlap of the statistically significant protein-disease associations using plasma *cis* pQTLs, plasma *cis* eQTLs and a Mendelian randomisation approach across four neurodegenerative diseases. The Venn diagrams show the number and the percentage of proteins significantly associated with each disease using plasma *cis* pQTLs or *cis* eQTLs, and their overlap, using a 5% false discovery rate. Each panel corresponds to a specific neurodegenerative disease: (A) Alzheimer's Disease, (B) Parkinson's Disease, (C) Amyotrophic Lateral Sclerosis, and (D) Multiple Sclerosis. The intensity of the red shading reflects the number of significant associations, with darker shades representing a greater number of associations.

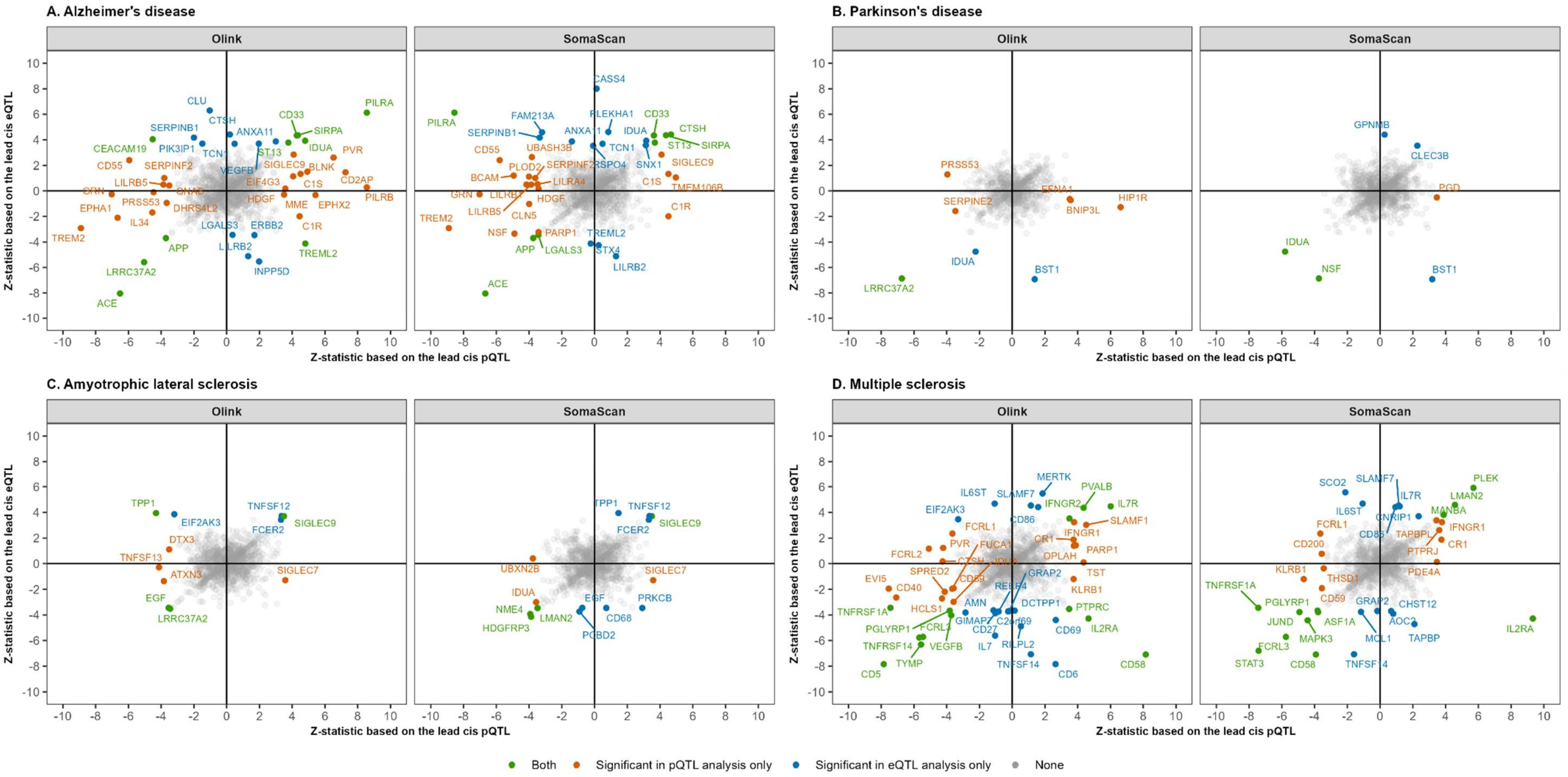

Supplementary Figure S6. Comparison of Z-statistics for the association of plasma protein abundance and plasma mRNA abundance with neurodegenerative diseases. Each panel corresponds to a specific neurodegenerative disease: (A) Alzheimer's Disease, (B) Parkinson's Disease, (C) Amyotrophic Lateral Sclerosis, and (D) Multiple Sclerosis. Proteins that were statistically significant in either the *cis* pQTL or *cis* eQTL analyses are annotated and color-coded.

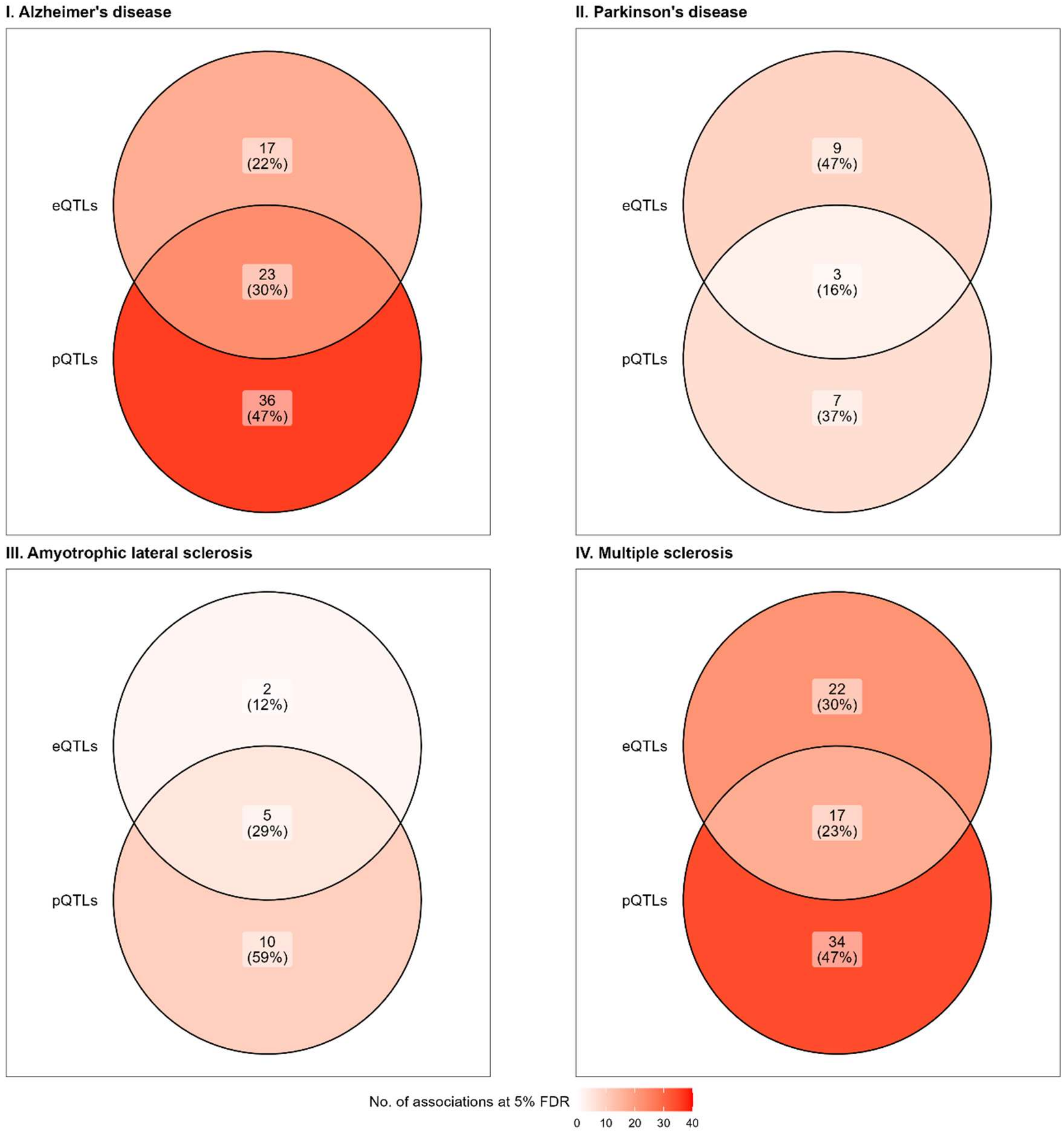

Supplementary Figure S7. Overlap of the statistically significant protein-disease associations using plasma *cis* pQTLs, cortex *cis* eQTLs and a Mendelian randomisation approach across four neurodegenerative diseases. The Venn diagrams show the number and the percentage of proteins significantly associated with each disease using plasma *cis* pQTLs or cortex *cis* eQTLs, and their overlap, using a 5% false discovery rate. Each panel corresponds to a specific neurodegenerative disease: (A) Alzheimer's Disease, (B) Parkinson's Disease, (C) Amyotrophic Lateral Sclerosis, and (D) Multiple Sclerosis. The intensity of the red shading reflects the number of significant associations, with darker shades representing a greater number of associations.

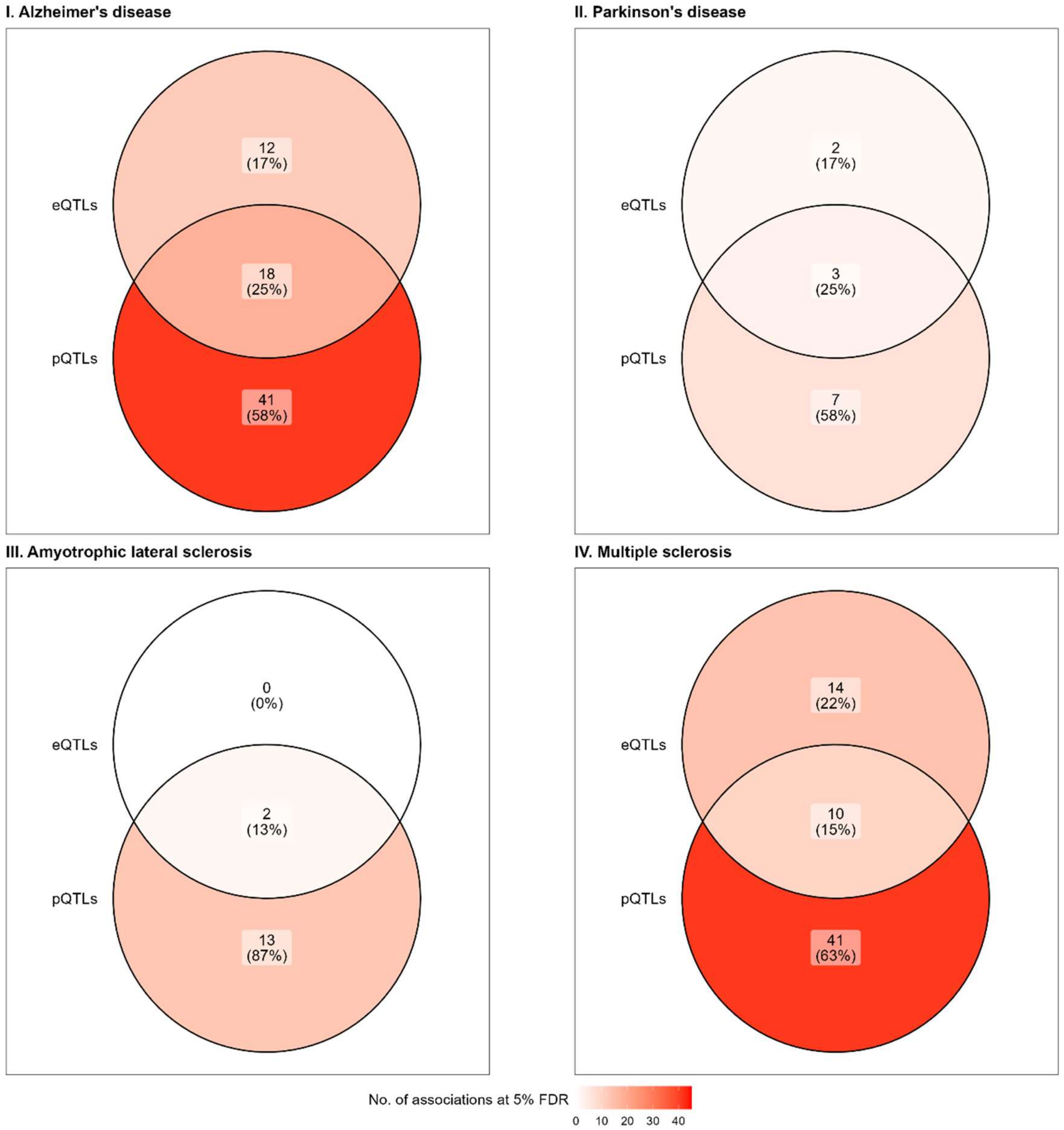

Supplementary Figure S8. Overlap of the statistically significant protein-disease associations using plasma *cis* pQTLs, cerebellum *cis* eQTLs and a Mendelian randomisation approach across four neurodegenerative diseases. The Venn diagrams show the number and the percentage of proteins significantly associated with each disease using plasma *cis* pQTLs or cerebellum *cis* eQTLs, and their overlap, using a 5% false discovery rate. Each panel corresponds to a specific neurodegenerative disease: (A) Alzheimer's Disease, (B) Parkinson's Disease, (C) Amyotrophic Lateral Sclerosis, and (D) Multiple Sclerosis. The intensity of the red shading reflects the number of significant associations, with darker shades representing a greater number of associations.

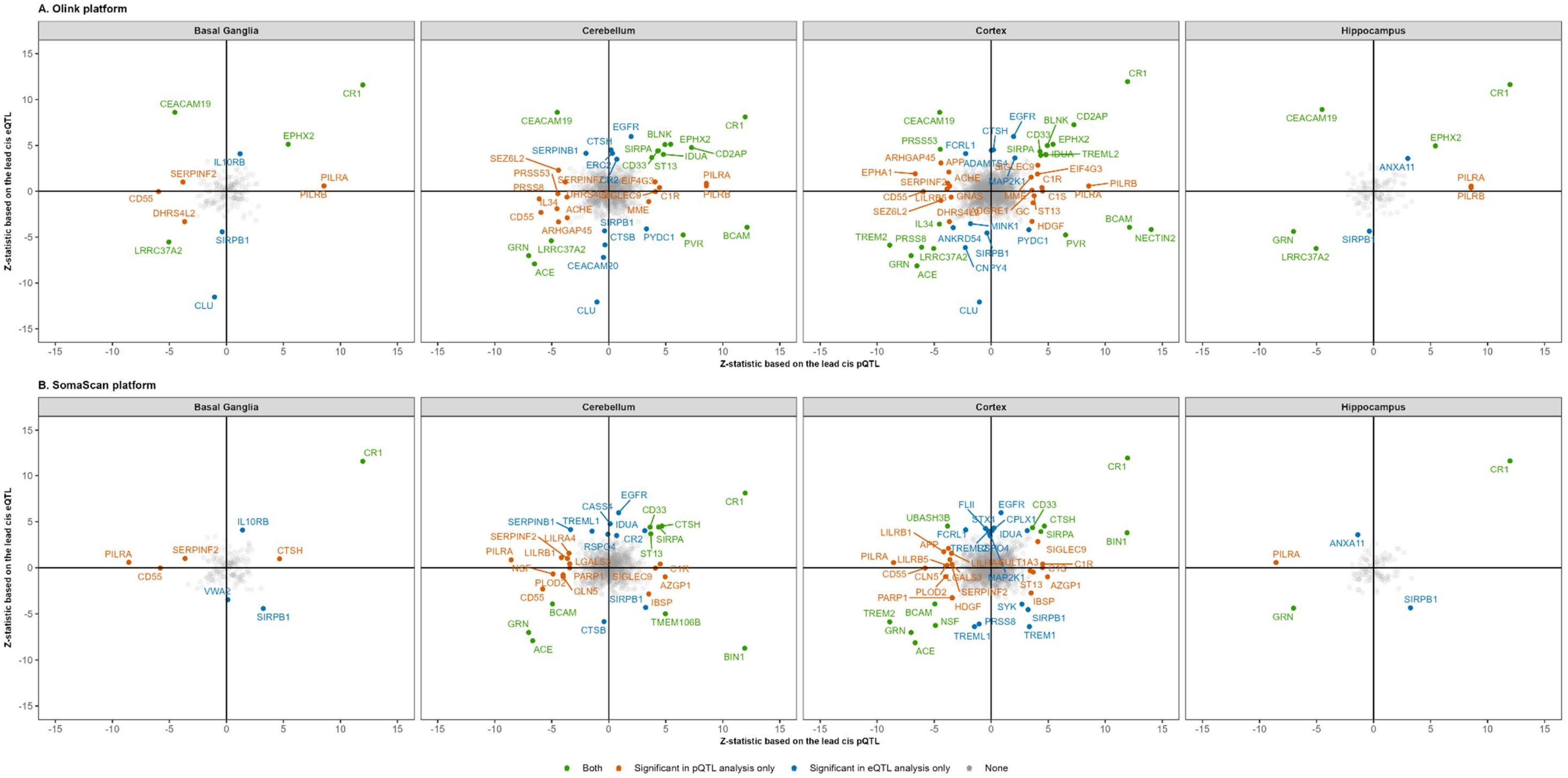

Supplementary Figure S9. Comparison of the Z-statistics for the association of plasma protein abundance and mRNA abundance in brain with Alzheimer's disease. Panels (A) and (B) represent results from the Olink and SomaScan platforms, respectively. Within each panel, different brain regions (Basal Ganglia, Cerebellum, Cortex, and Hippocampus) are shown as separate facets. Proteins that were statistically significant in either the *cis* pQTL or *cis* eQTL analyses are annotated and color-coded.

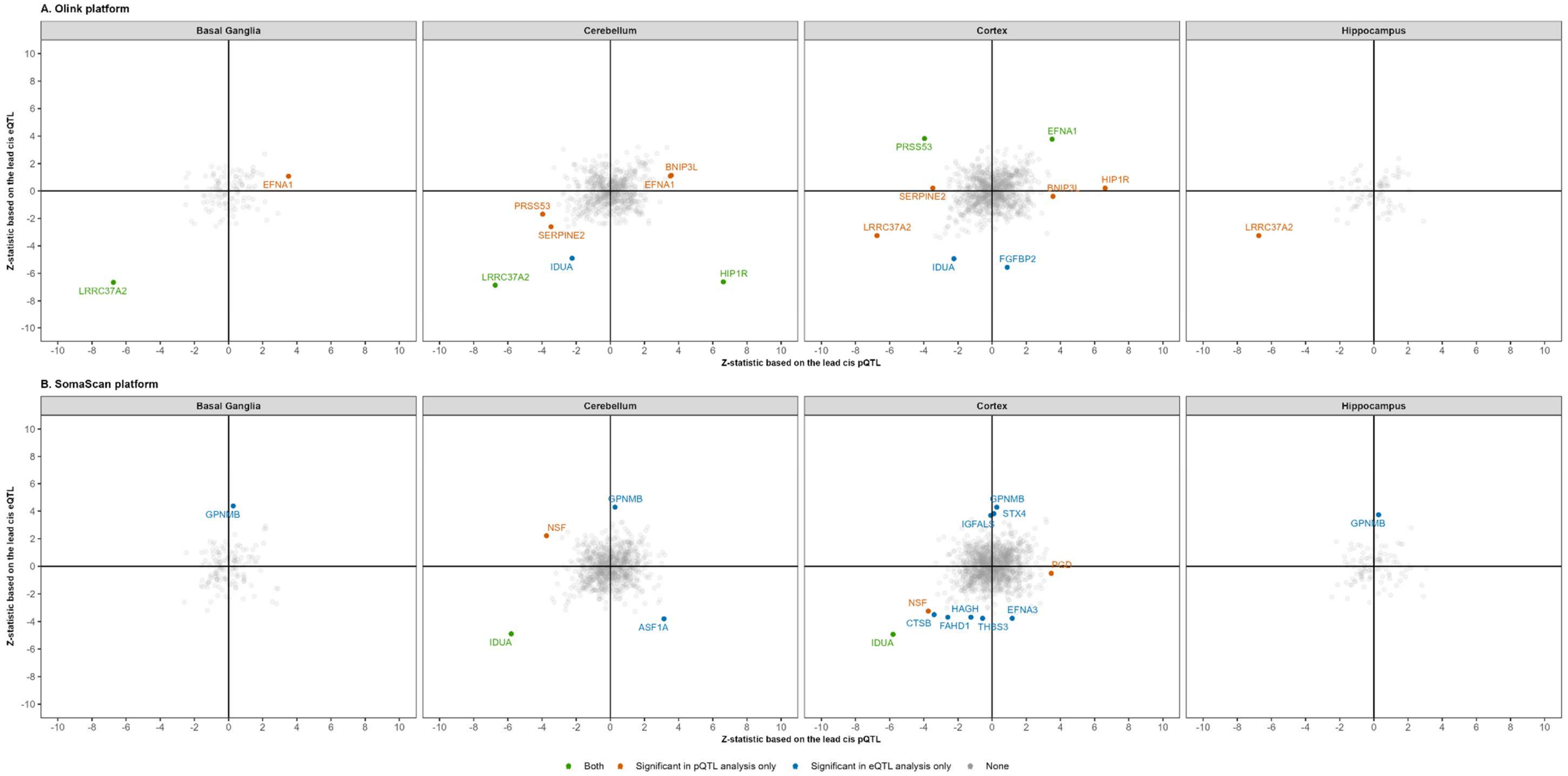

Supplementary Figure S10. Comparison of the Z-statistics for the association of plasma protein abundance and mRNA abundance in brain with Parkinson's disease. Panels (A) and (B) represent results from the Olink and SomaScan platforms, respectively. Within each panel, different brain regions (Basal Ganglia, Cerebellum, Cortex, and Hippocampus) are shown as separate facets. Proteins that were statistically significant in either the *cis* pQTL or *cis* eQTL analyses are annotated and color-coded.

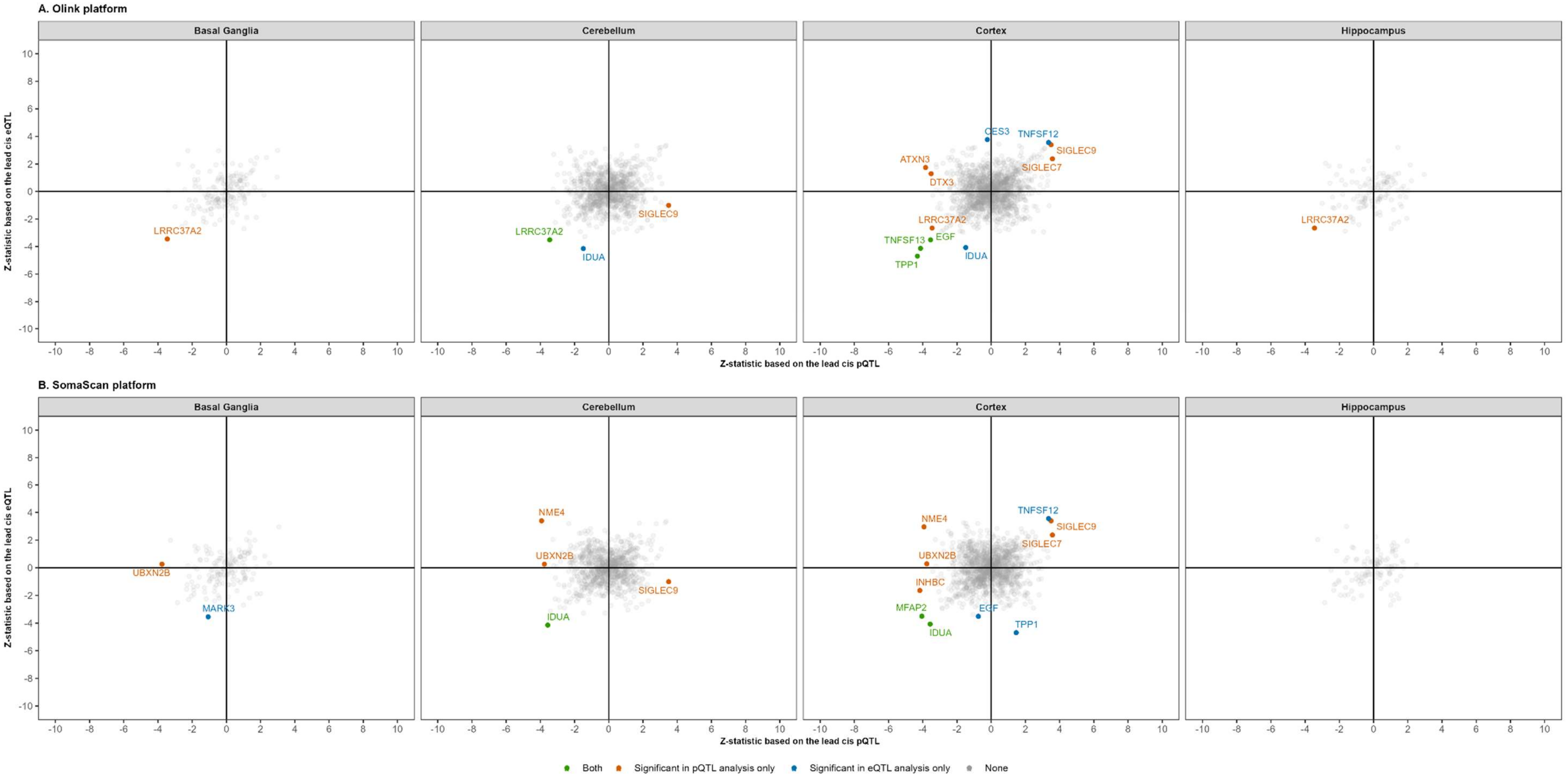

Supplementary Figure S11. Comparison of the Z-statistics for the association of plasma protein abundance and mRNA abundance in brain with amyotrophic lateral sclerosis. Panels (A) and (B) represent results from the Olink and SomaScan platforms, respectively. Within each panel, different brain regions (Basal Ganglia, Cerebellum, Cortex, and Hippocampus) are shown as separate facets. Proteins that were statistically significant in either the *cis* pQTL or *cis* eQTL analyses are annotated and color-coded.

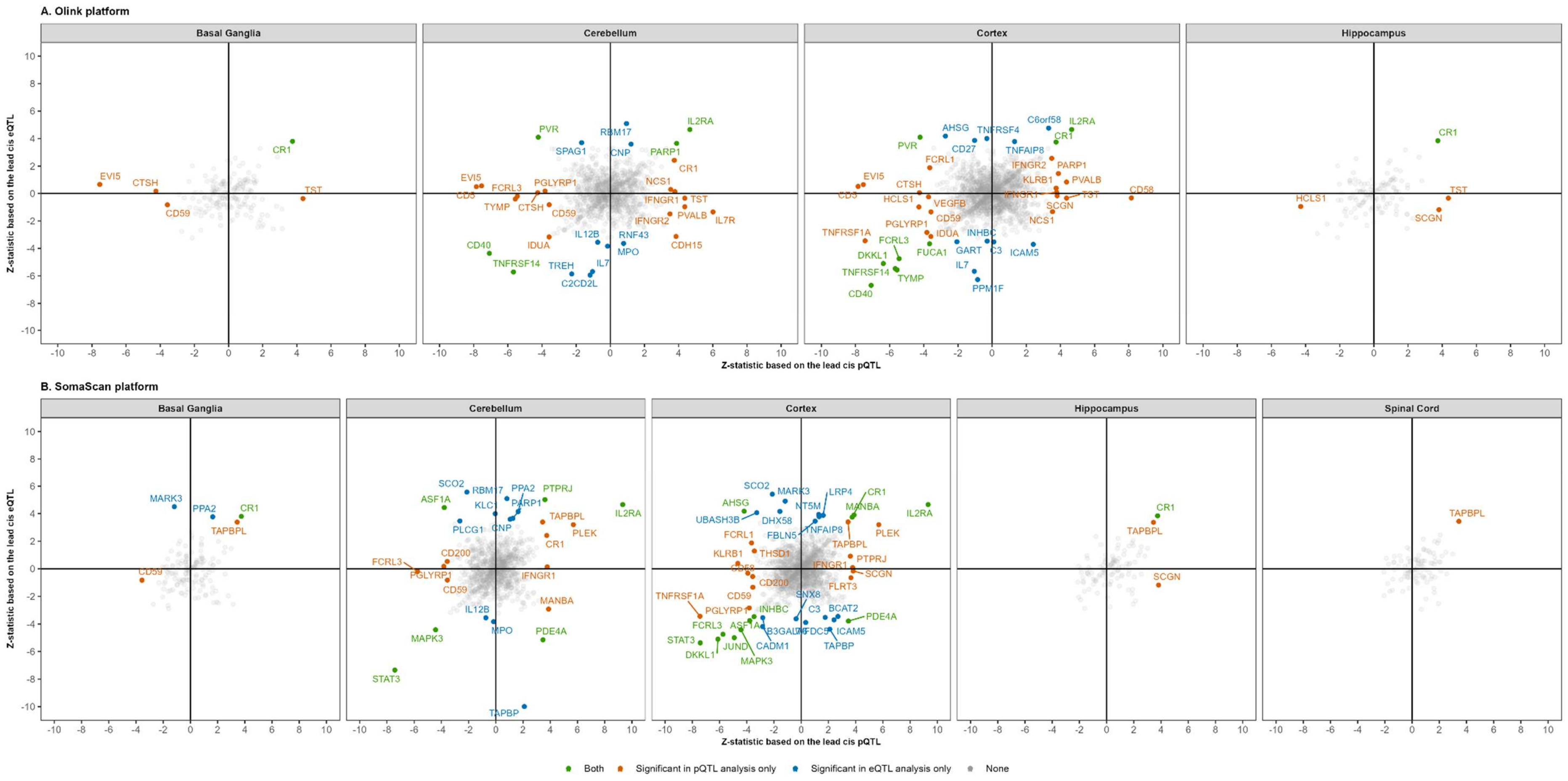

Supplementary Figure S12. Comparison of the Z-statistics for the association of plasma protein abundance and mRNA abundance in brain with multiple sclerosis. Panels (A) and (B) represent results from the Olink and SomaScan platforms, respectively. Within each panel, different brain regions (Basal Ganglia, Cerebellum, Cortex, and Hippocampus) are shown as separate facets. Proteins that were statistically significant in either the *cis* pQTL or *cis* eQTL analyses are annotated and color-coded.

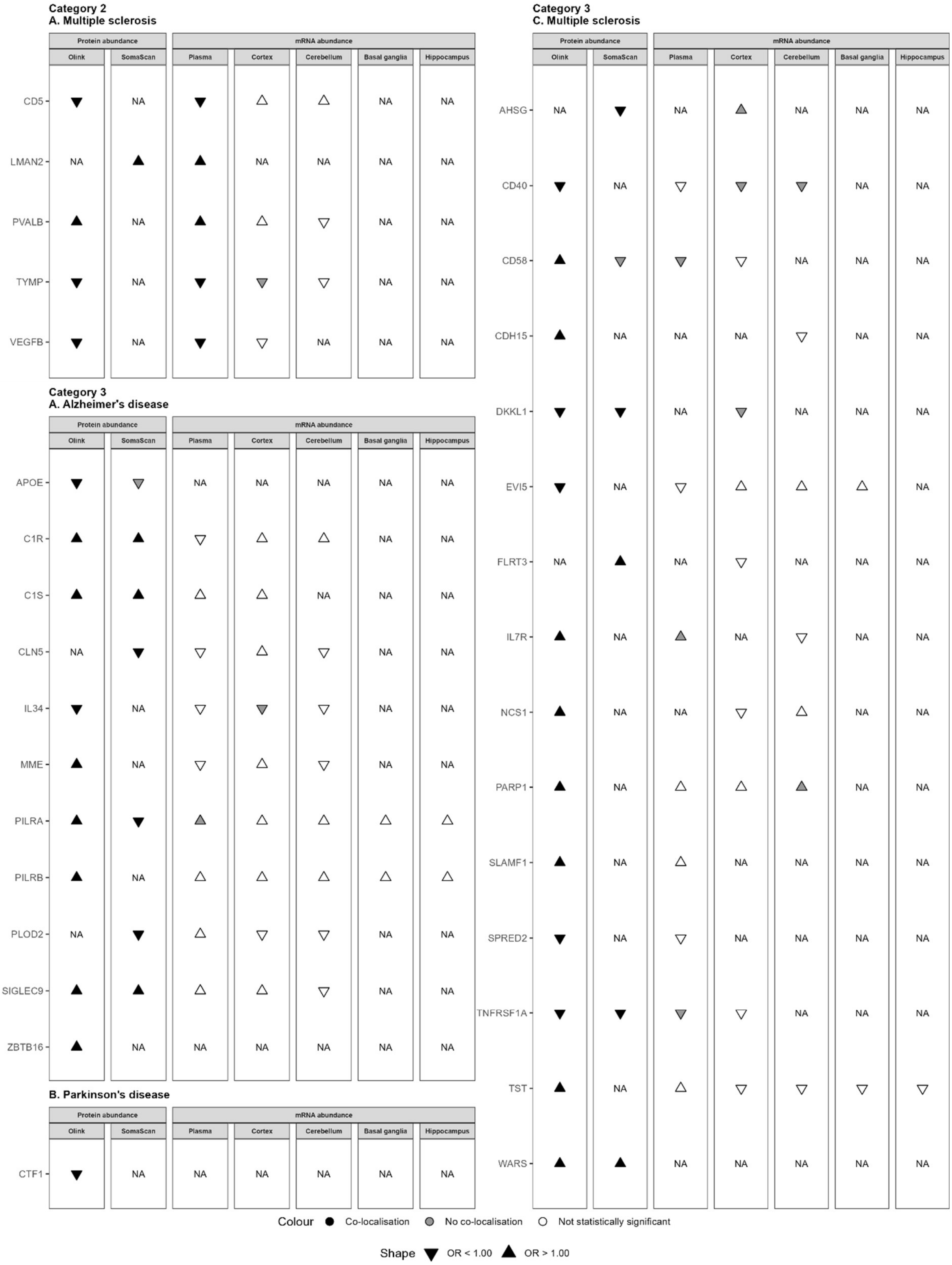

Supplementary Figure S13. Summary of cumulative evidence using plasma *cis* pQTLs and plasma or brain *cis* eQTLs as instrumental variables. Each panel corresponds to one neurodegenerative disease: (A) Alzheimer's Disease, (B) Parkinson's Disease, (C) Amyotrophic Lateral Sclerosis, and (D) Multiple Sclerosis. Category 2 includes protein-disease

associations supported both Mendelian randomisation and co-localisation using the lead plasma *cis* pQTL and the lead plasma *cis* eQTL (but without support by *cis* brain eQTLs). Category 3 includes protein-disease associations supported by both Mendelian randomisation and co-localisation using the lead plasma *cis* pQTL (but without support by *cis* plasma or brain eQTLs). When the Mendelian randomisation analysis did not show a statistically significant effect, co-localisation was not performed. The notation NA indicates cases where a cis pQTL or a cis eQTL was not available for a particular protein.

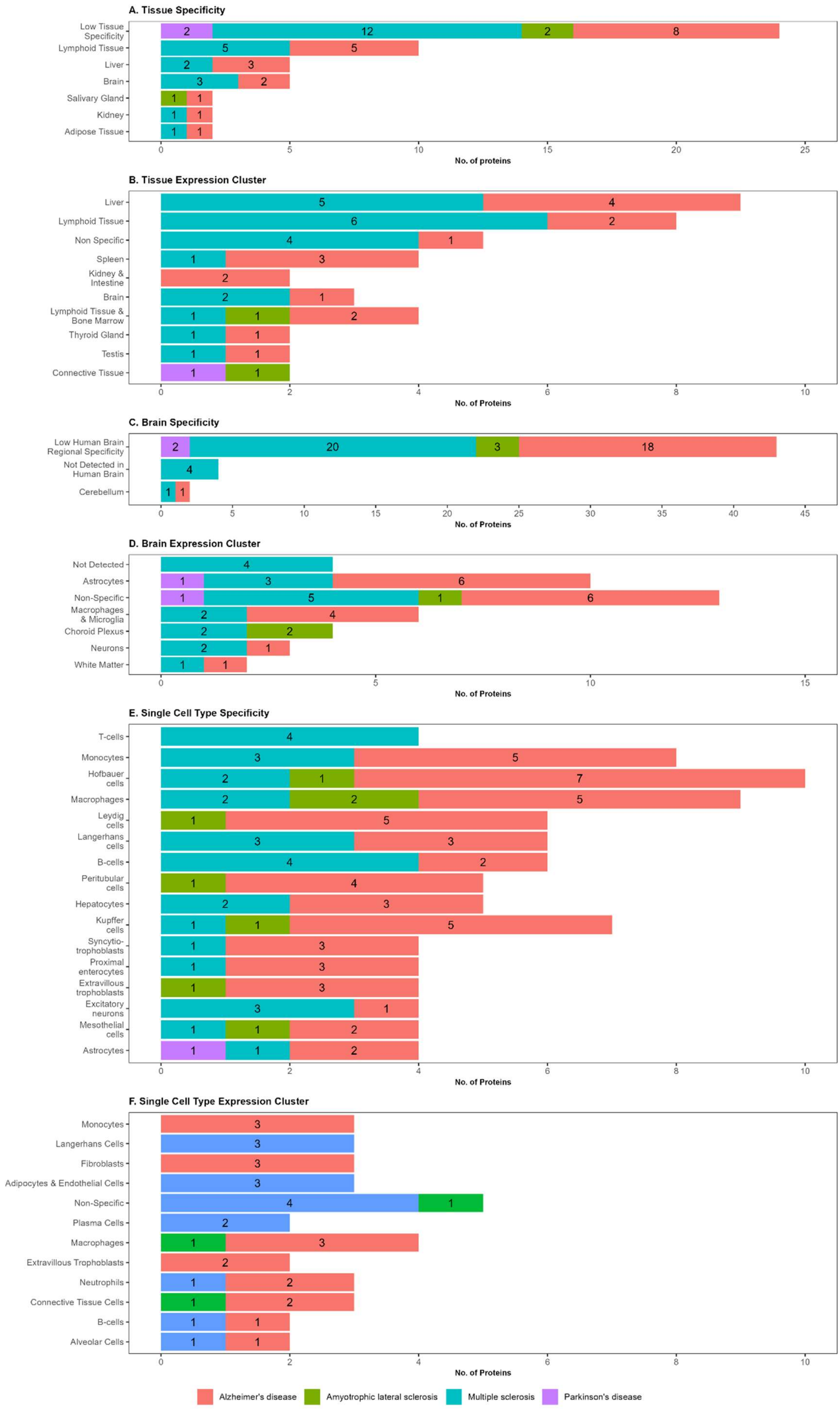
A. Tissue Specificity
Low Tissue Specificity
Lymphoid Tissue
Liver
Brain
Salivary Gland
Kidney
Adipose Tissue
No. of proteins
B. Tissue Expression Cluster
Liver
Lymphoid Tissue
Non Specific
Spleen
Kidney & Intestine
Brain
Lymphoid Tissue & Bone Marrow
Thyroid Gland
Testis
Connective Tissue
No. of Proteins
C. Brain Specificity
Low Human Brain Regional Specificity
Not Detected in Human Brain
Cerebellum
No. of Proteins
D. Brain Expression Cluster
Not Detected
Astrocytes
Non-Specific
Macrophages & Microglia
Choroid Plexus
Neurons
White Matter
No. of Proteins
E. Single Cell Type Specificity
T-cells
Monocytes
Hofbauer cells
Macrophages
Leydig cells
Langerhans cells
B-cells
Peritubular cells
Hepatocytes
Kupffer cells
Syncytio-trophoblasts
Proximal enterocytes
Extravillous trophoblasts
Excitatory neurons
Mesothelial cells
Astrocytes
No. of Proteins
F. Single Cell Type Expression Cluster
Monocytes
Langerhans Cells
Fibroblasts
Adipocytes & Endothelial Cells
Non-Specific
Plasma Cells
Macrophages
Extravillous Trophoblasts
Neutrophils
Connective Tissue Cells
B-cells
Alveolar Cells
No. of Proteins
Alzheimer's disease
Amyotrophic lateral sclerosis
Multiple sclerosis
Parkinson's disease

Supplementary Figure S14. Tissue, brain, and single cell specificity and expression clusters for proteins associated with neurodegenerative diseases. Data are derived from the *Human Protein Atlas* and are available in the Supplementary Table S12. The results are presented by neurodegenerative disease which is colour-coded. To improve the visual clarity of the figure, only tissues with at least two proteins are shown in panel (B), and only cell types with at least four proteins are shown in panel (E) and three proteins in panel (F).
